# Insight into the phylogeny and responses of species from the genus *Sergia* (Campanulaceae) to the climate changes predicted for the Mountains of Central Asia (a world biodiversity hotspot)

**DOI:** 10.1186/s12870-024-04938-4

**Published:** 2024-04-01

**Authors:** Lizaveta Vintsek, Ewelina Klichowska, Arkadiusz Nowak, Marcin Nobis

**Affiliations:** 1 Institute of Botany, Faculty of Biology, Jagiellonian University, Gronostajowa 3, Kraków, 30–387 Poland; 2 Polish Academy of Sciences Botanical Garden, Center for Biological Diversity Conservation in Powsin, Prawdziwka 2, Warsaw, 02-973 Poland; 3 Botanical Garden of the Wrocław University, Sienkiewicza 23, 50-335 Wrocław, Poland

**Keywords:** Bell-flowers, Climate warming, DArT sequencing, Genome-wide analysis, Niche modelling, Phylogeny

## Abstract

**Background:**

Together with other elevated areas, the Mountains of Central Asia are significantly threatened by ongoing climate change. The presence of refuges during the glaciations makes the region extremely rich in species, especially endemic ones. However, the limited potential for colonisation of other habitats makes rocky-related species with ‘island‐like’ distribution, particularly vulnerable to climate change. To understand the processes underlying species response to climate warming, we assessed differences in ecological niches and phylogenetic relationship of two geographically disjunctive alpine species belonging to the genus *Sergia*. The taxa are considered Tertiary relicts, endemic to the Tian Shan and Pamir-Alai Mountains. To illustrate range dynamics and differences in occupied niches of *Sergia* species, we used Ecological Niche Modelling of current and future distribution. Whereas, to reconstruct the phylogenetic relationship within and between *Sergia* and other related Campanulaceae species from the region we used molecular data (ITS, cpDNA, DArTseq-derived SNPs).

**Results:**

The results reveal that the genus *Sergia* is a polyphyletic group, and its representatives differ geographically, ecologically and genetically. Both *S. regelii* and *S. sewerzowii* constitute a common clade with *Asyneuma* group, however, *S. sewerzowii* is more closely related to *Campanula alberti* (a species that has never previously been considered closely related to the genus *Asyneuma* or *Sergia*) than to *S. regelii*. *Sergia sewerzowii* is adapted to lower elevations with higher temperatures, while *S. regelii* prefers higher elevations with lower temperatures. The future distribution models demonstrate a dramatic loss of *S. regelii* range with a shift to suitable habitats in higher elevations, while the potential range of *S. sewerzowii* increases and shifts to the north.

**Conclusions:**

This study shows that *S. regelii* and *S. sewerzowii* have a long and independent evolution history. *Sergia regelii* and *S. sewerzowii* significantly differ in realised niches. These differences are mirrored in the response of the studied endemics to future climate warming. As suitable habitats shrink, rapid changes in distribution can lead to species' range loss, which is also directly related to declines in genetic variability. The outcomes of this paper will help to more precisely assess the impact of climate changes on rocky-related plant species found in this world’s biodiversity hotspot.

**Supplementary Information:**

The online version contains supplementary material available at 10.1186/s12870-024-04938-4.

## Background

In this century, climate change is one of the strongest factors affecting biodiversity loss as well as largely determining the geographical distribution of species [1, 2], their phenology, physiology, community structures, and ecosystem functions [3]. The most rapid species response to climate changes is observed in alpine regions in the form of altitudinal range shifts [4]. Along with increasing temperature, cold-adapted mountain species are forced to move to cooler areas, which in this case means to the north or higher elevations, depending on their dispersal capacities and the availability of suitable habitats [5–7]. In extreme cases, upslope movement results in species extinction because they reach the highest elevations and have no place to escape [8].

The Mountains of Central Asia known also as a biodiversity hotspot, is one of the world’s major centers of plant diversity [9] and among the most elevated areas highly susceptible to climate change [10, 11]. The region is extremely rich in species due to a complex of highly heterogeneous and isolated habitats with diverse topographies, soil types, and microclimates relating to altitude, slope exposure, and precipitation [12–14]. In addition, the considerable richness of taxa is linked to the fact that during the Quaternary glaciations, ice sheets did not reach the Mountains of Central Asia, and local mountain glaciers did not cover the patches of all ecosystems, saving areas that have become refuges for Tertiary flora [15].

Among the most interesting habitats in the mountains of Central Asia are rocky habitats, which include vertical rock faces, fissures, clefts, crevices, and rock ledges. They are characterised by great edaphic drought as a result of the soil’s reduced capacity for water retention [16]. Rocky habitats may also differ from each other considerably in terms of humidity, type of substrate rock, insolation, temperature, and inclination [17]. It is an ‘island‐like’ environment, presenting discrete patches of habitats surrounded by strongly contrasting areas in terms of environmental conditions. The harsh environment and patchy structure of chasmophytic habitats make it a suitable biotope for many endemic and specialised plant taxa with narrow niches [18, 19]. The considerable structural differences of chasmophytic flora are highlighted by including the communities of rocky habitats into a separate class named *Asplenietea trichomanis* [20, 21], with the *Campanuletalia incanescentis* order of phytocoenoses distinguished within the chasmophytic vegetation in the Mountains of Central Asia [22–25]. The limited potential for the colonisation of other habitats makes rocky vegetation particularly vulnerable to changing environmental conditions [26], and thus, there is an urgent need to estimate the impact of climate change on chasmophytic plants by creating models of its future potential distribution [3].

The genus *Sergia* Fed. comprises two geographically disjunctive, alpine plant species associated with rocky habitats, *S. regelii* (Trautv.) Fed. and *S. sewerzowii* (Regel) Fed. These species are considered Tertiary relicts, endemic to the Mountains of Central Asia [27, 28]. *Sergia regelii* occurs mainly in the western Pamir-Alai Mountains (NW Tajikistan, SE Uzbekistan, and E Turkmenistan) in fissures, crevices and ledges within steep rock walls, stones, and loamy soils at an altitude between (1400-)1600 and 2400(-3200) m [29, 30] (Supplementary Material Fig. S1). *Sergia sewerzowii* also occurs in similar habitats, but is distributed in the north-western Tian Shan Mountains (SE Karatau and Talass Alatau ridges in S Kazakhstan) at an altitude of 600–1400(-2000) m (Supplementary Material Fig. S1). Although the two taxa are placed in the genus *Sergia*, they differ considerably in distribution and morphology. *Sergia regelii* is generally characterised by taller stems and flowers (petals, calyx, sepals) and densely hairy leaves, whereas *S. sewerzowii* is a smaller and glabrous plant (Supplementary Material Table S1). Although phylogenetic studies of the Campanulaceae family have a long tradition [26, 30–34], to our knowledge, with the exception of the work of Xu and Hong [35], representatives of the genus *Sergia* have not been examined. Based on the combined dataset of six chloroplast regions, Xu and Hong [35] presented a phylogenetic tree, in which the genus appears to be non-monophyletic, similarly as most of the other genera analysed within Campanulaceae analysed with using a molecular approach [31–34]

*Sergia regelii* and *S. sewerzowii* have not been studied in detail in terms of phylogeny and ecology, although they belong to the most beautiful and narrow-range group of chasmophytic plants, threatened in the face of future climate change. To better understand the processes underlying species response to future climate change we will combine the results of phylogenetic analyses and Ecological Niche Modelling of those two endemic representatives of the genus *Sergia*. In particular, we would like to (i) verify the hypothesis of the monophyly of the genus *Sergia* and analysis of the phylogenetic relations with the other Campanulaceae species occurring in the mountains of Central Asia; (ii) define the differences in realised niches and predict the spatial and temporal patterns of *S. regelii* and *S. sewerzowii* distribution; as well as (iii) evaluate the vulnerability of these alpine species to predicted climate change. The findings will contribute to a better understanding of the climatic factors affecting the distribution of chasmophytic endemics and provide a theoretical basis for their conservation and restoration purposes by identifying areas susceptible to climate change and establishing unified conservation strategies aimed at preserving the extraordinary rates of endemism of this mountain biodiversity hotspot.

## Results

### Chloroplast and ribosomal DNA variation

The total alignment of ITS across 12 analysed individuals (including species belonging to *Asyneuma* Griseb. & Schenk, *Sergia* and *Campanula alberti* Trautv.) was 683 bp. The alignment revealed differences in sequence length between the studied samples. The sequences only slightly differ in length and range, from 678 bp (*Campanula alberti* Trautv.) up to 682 bp (*Asyneuma argutum* (Regel) Bornm. and *S. sewerzowii*). The alignment contains 71 parsimony informative sites (67 with two variants and 4 with three variants), 3 singleton variable sites and 9 indel (insertion/deletion) events. We failed to amplify the Astho3 sample of *A. thomsonii* (Hook.f.) Bornm. for the *pet*D region, Asarg2, Astra8, Sesew4, Sereg5 samples (*A. argutum*, *A. trautvetteri* (B.Fedtsch.) Bornm*.*, *S. sewerzowii* and *S. regelii* respectively) for *psb*D*-trn*T as well as part (in one direction) of Astra8 of *A. trautvetteri* for *trn*K*-psb*A (for geographical origin of the samples see Supplementary Material Table S2). The concatenated alignment of *pet*D, *trn*K*-psb*A, *rpl*16, *psb*D*-trn*T, and *trn*S-*trnf*M regions for the 12 studied individuals of *Asyneuma*, *Sergia* and *C. alberti*, without an outgroup, was 6385 bp. The concatenated alignment contains 102 parsimony informative sites with two variants, 5 singleton variable sites and 89 insertion/deletions.

### Phylogenetic relationships

**Fig. 1:**
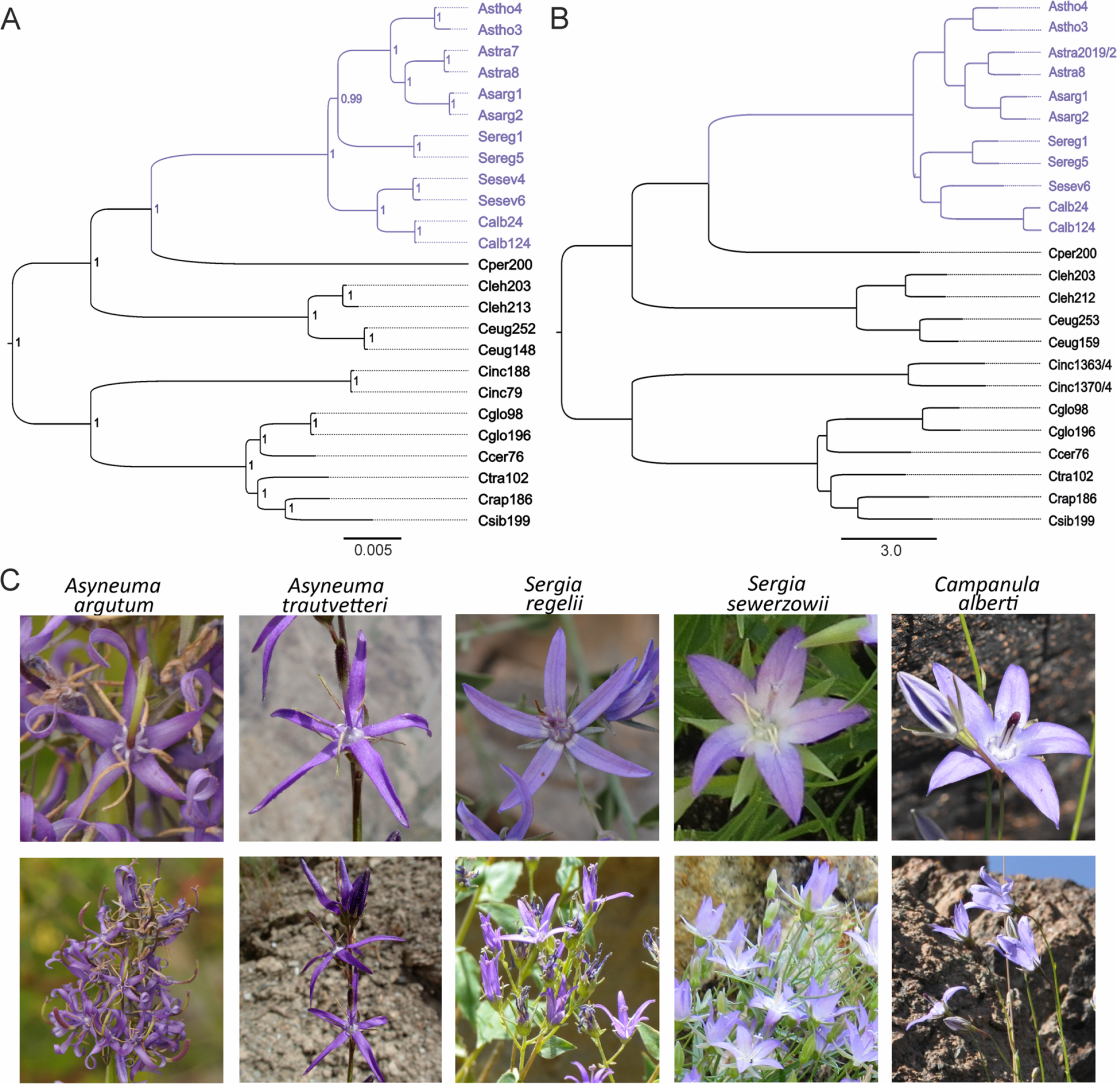
Phylogenetic relationship and similarity in flower morphology of the studied group of species. **A** Bayesian Inference of phylogeny based on concatenated cpDNA data (25 individuals, 6710 bp); numbers at the nodes indicates the Bayesian posterior probability values, **B** Neighbor Joining tree based on DArTseq derived SNPs (24 individuals, 1047 SNPs), **C** Flower morphology. Photographs of *A. argutum*, *A. trautvetteri*, *C. alberti* and *S. regelii* by M. Nobis & A. Nowak, photograph of *S. sewerzowii* by A. Ebel. An explanation of abbreviations of samples used in analyses is given in the Supplementary Material Table S2

The trees revealed by the Bayesian Inference (BI) based on cpDNA as well as Neighbor-Joining (NJ) based on DArTseq derived SNPs yielded similar topologies (Fig. 1A, B), showing clear and well-supported (in the case of BI) divergence between both studied *Sergia* species. In all trees, samples of *C. alberti* group together with samples of *S. sewerzowii*, indicating that the taxa split from a common ancestor. The current range of *S. sewerzowii* and *C. alberti* partially overlap and are characterised by an altitudinal disjunction, where *C. alberti* prefers higher-situated habitats (Supplementary Material Fig. S2).

In all analyses, both *Sergia* species as well as *C. alberti* form a common clade with representatives of the genus *Asyneuma*. Analyses revealed considerable uncertainty in the case of *S. regelii* (Sereg1, Sereg5), which was assigned either as a sister group to the *Asyneuma* (Asarg1, Asarg2, Astho3, Astho4, Astra7, Astra8) or *Sergia sewerzowii*-*Campanula alberti* group (Sesew6, Calb24, Calb124) (Fig. 1 A, B). Only the ITS analysis indicates that both species of *Sergia* share the common ancestor (Supplementary Material Fig. S3), however in this analysis, particular clades referring to *Sergia*, *Campanula alberti* and *Asyneuma* are organised in a polytomy.

### Performance of the models and potential current species distribution

**Fig. 2:**
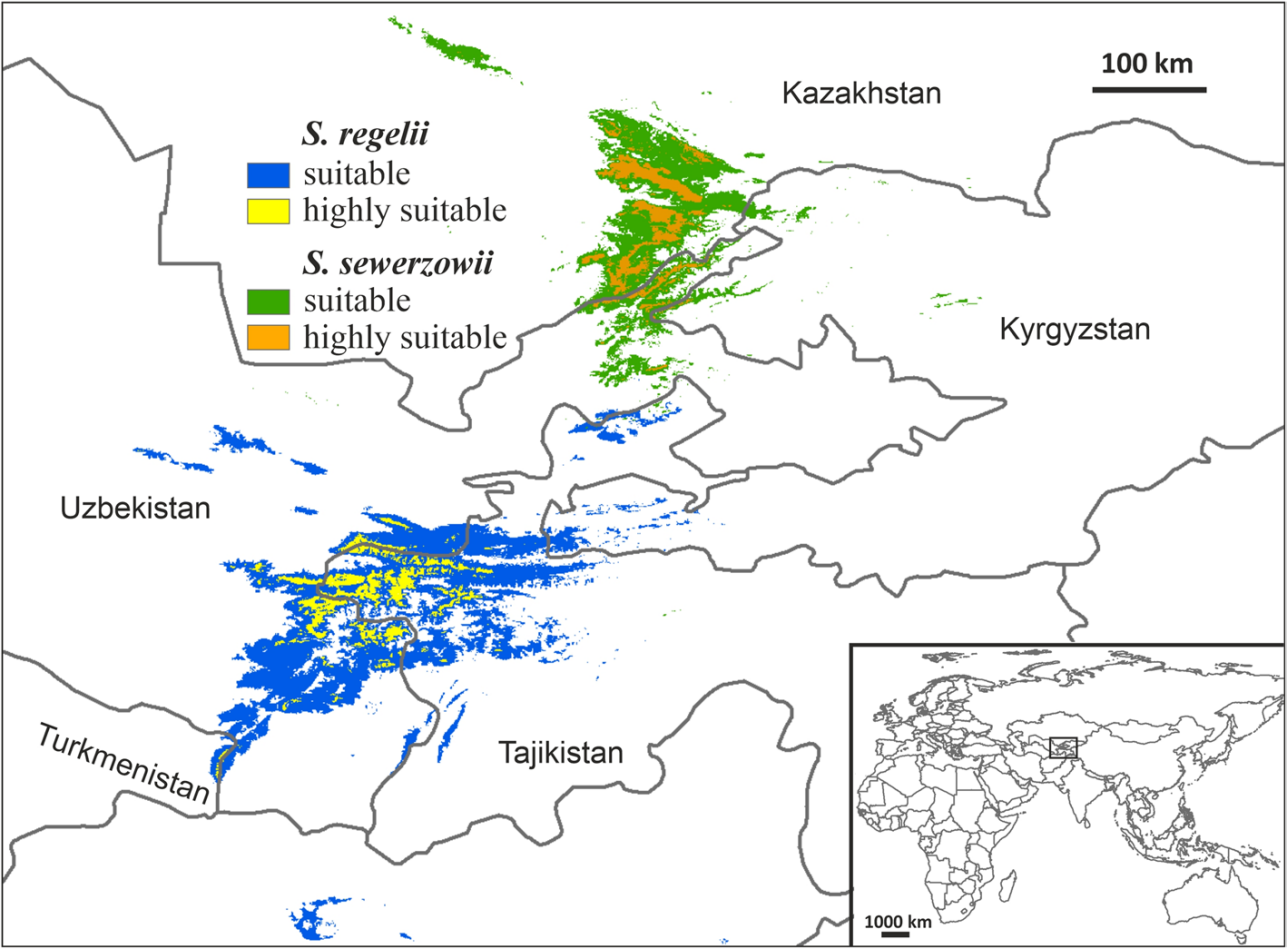
Potential current distribution of *S. regelii* and *S. sewerzowii*. The present suitable area is defined as an area with a probability of species presence higher than the Equal test sensitivity and specificity logistic threshold. A highly suitable area is one with a probability of presence from 0.6 to 1

The values of average test AUC obtained from models were high (0.991 ± 0.003 for *S. regelii* and 0.989 ± 0.012 for *S. sewerzowii*), which confirms high levels of the model’s predictive performance. According to the results of Maxent modelling, variables bio7, srh, bio19, crf, cec, and bio17 were the major contributors to the distribution model of *S. regelii* (Table 1), with a cumulative contribution of 97.6%. In the case of *S. sewerzowii*, the main contributors were bio19, srl, bio17, snd, crf and elev. Their cumulative contribution was 98.4%. The suitable area for *S. regelii* (probability of presence > 0.1313) was 27,226 km^2^ (Fig. 2). The 3,658 km^2^ (13.4% of the total suitable area) was identified as area of high probability of occurrence (0.6–1). In the case of *S. sewerzowii,* the suitable area was almost twice time lower, 14,554 km^2^ (probability of presence > 0.2263), however the area of high potential suitability (0.6–1) was 2,989 km^2^, which accounted for over 20% of the total suitable area.

**Table 1:** Contribution of environmental variables used for modelling the potential distribution of *S. regelii* and *S. sewerzowii*

Code	Environmental variables	% contribution	
		*S. regelii*	*S. sewerzowii*
bio7	Temperature Annual Range (BIO5-BIO6), °C	60	0.4
bio8	Mean Temperature of Wettest Quarter, °C	0.3	0.9
bio17	Precipitation of Driest Quarter, mm	1.6	14.6
bio19	Precipitation of Coldest Quarter, mm	9.3	35.1
elev	Elevation, m	-	3.7
srl	Average solar radiation in a quarter with lowest solar radiation, W/m2	0.2	30.7
srh	Average solar radiation in a quarter with highest solar radiation, W/m2	22.1	-
cec	Cation Exchange Capacity of soil, cmolc/kg	2	0
ocs	Soil organic carbon stock, t/ha	1.2	0.2
pH	pH index measured in water solution	0.7	0.1
crf	Volumetric percentage of coarse fragments (> 2 mm), %	2.6	5.6
snd	Weight percentage of sand particles (0.05–2 mm), %	-	8.7

The current potential distribution of *S. regelii* was predicted in the Pamir-Alai Mts, particularly in the Kuhitang, Baisun-tau, Hissar, Turkestan, and Zeravshan ridges, from where its occurrence was already known (Supplementary Material Fig. S1). These results confirm the accuracy of the model because the potential and actual distribution of *S. regelii* significantly overlap. In the case of the potential distribution of *S. sewerzowii*, besides known localities in the Karatau and Talas Alatau ridges (south Kazakhstan), suitable habitat was also predicted in east Uzbekistan, from where the species has not been listed yet.

### Comparison of ecological niches

**Fig. 3:**
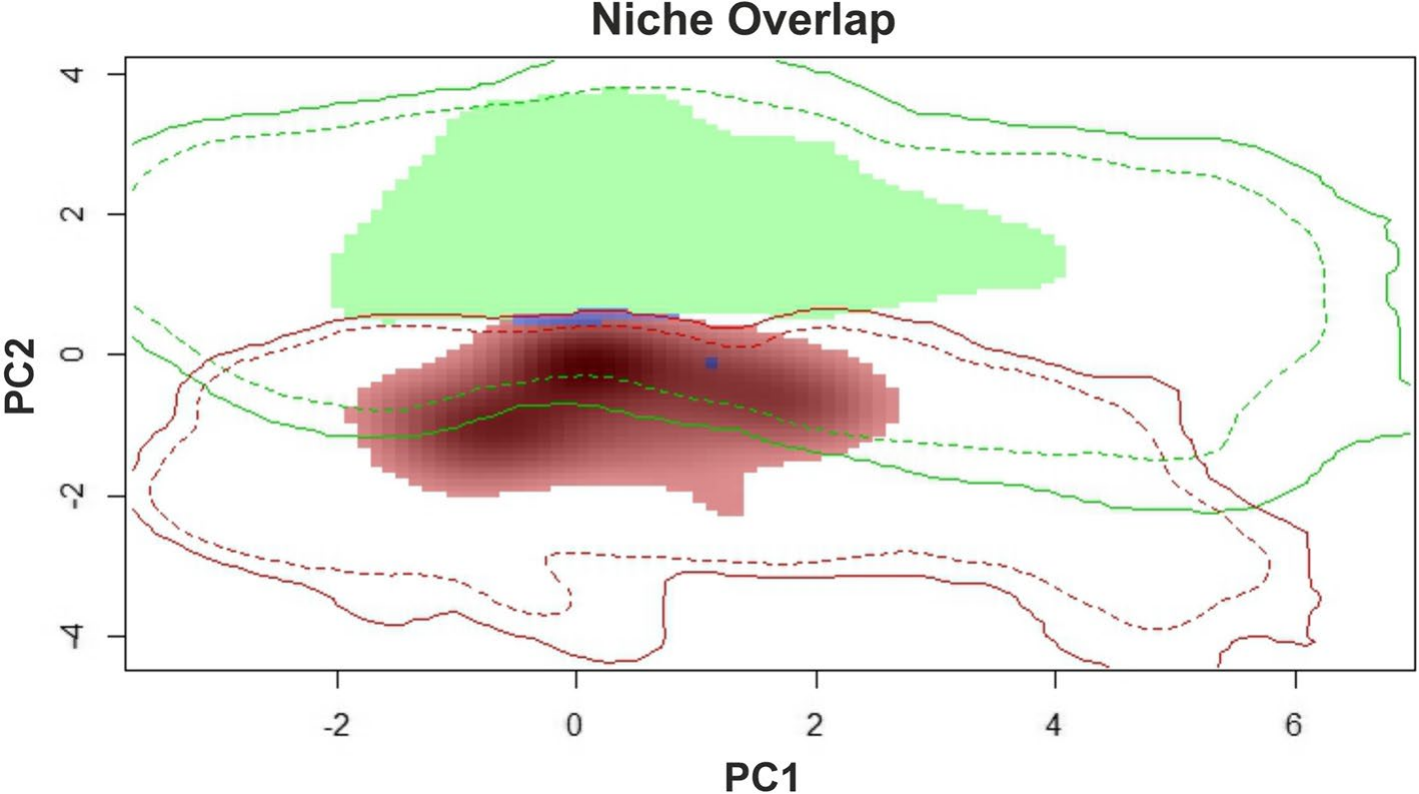
PCA of niche overlap (green shading – realised niche of *S. regelii*, red shading – realised niche of *S. sewerzowii*, blue shading – niches overlap). Solid and dashed lines – 100% and 75% of the available (background) environment for *S. regelii* (green) and *S. sewerzowii* (red)

The PCA biplot shows that the realised niches of *S. regelii* and *S. sewerzowii* slightly overlap (Fig. 3). Among the analysed variables, bio7, bio8, bio19, srl, and cec differentiate the niches of the examined species the most (Supplementary Material Fig. S4). The cumulative contribution of these variables according to the Maxent model is 71.8% for *S. regelii* and 67.1% for *S. sewerzowii*. Despite significant overlap of the other environmental variables (e.g. bio17, crs, ocs, and pH), the realised niches of the studied species differ noticeably.

Schoener’s D index and the I statistic are 0.0085 and 0.0505, respectively, reflecting a low level of niche similarity (Supplementary Material Table S3). The test for niche divergence (alternative = “lower”) shows significant distinctions between the *S. regelii* and *S. sewerzowii* niches, indicating that they are less equivalent than expected by chance (*p* < 0.05). The niche conservatism test (alternative = “greater”) indicates that the studied species’ niches are less equivalent (similar) than expected by chance (*p* > 0.05). Therefore, we can conclude that there is no significant niche conservatism between *S. regelii* and *S. sewerzowii*, however, there is significant niche divergence between them.

### Potential future species distribution under different scenarios of global warming

**Fig. 4:**
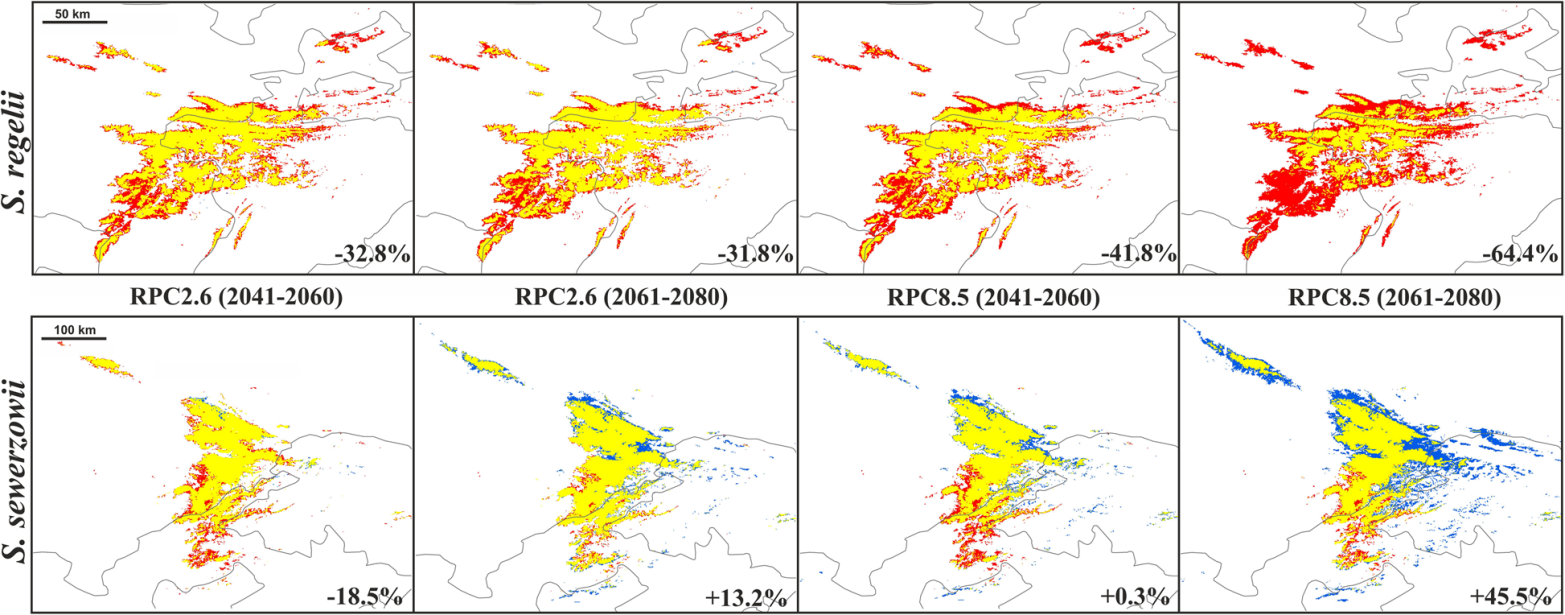
Changes in the potential niche distribution of *S. regelii* and *S. sewerzowii* during periods 2041–2060 and 2061–2080 under different climate change scenarios (blue – gain, red – loss, yellow – unchanged). Numbers in the left angles – percent of change in the potential distribution area compared with current distribution

The projected future models for both 2.6 and 8.5RCP emissions scenarios showed a progressive reduction of the extent of suitable areas of occurrence for *S. regelii*, in comparison to the potential current distribution (Supplementary Material Table S4, Fig. 4). According to the ‘optimistic’ emissions scenario (+ 2.6 W/km^2^), up to 2060 the potentially suitable areas for *S. regelii* will decrease by 32.8% and up to 2080, by 41.8%. Considering a ‘pessimistic’ scenario (+ 8.5 W/km^2^), the predicted reduction of suitable areas is even more significant (especially in the later period): 31.8% by 2060 and 64.4% by 2080. Changes in the potential geographic distribution of *S. sewerzowii* up to 2060 under the ‘optimistic’ emissions scenario start with habitat loss along the southern border of the distribution and gradually move to the north by 2080. According to ‘pessimistic’ scenario, *S. sewerzowii* will expand its range by 45.5% up to 2080. As in the optimistic scenario, the species may find new suitable niches north of its current range (Supplementary Material Fig. S5).

## Discussion

In the face of ongoing climate change and progressive habitat decrease, understanding the range dynamics of alpine plants vulnerable to extinction, restricted today to mid- and lower-elevated regions, is of pivotal importance [13]. The chasmophytic vegetation of Central Asian mountains consists of many endemics, significantly differing from other types of phytocoenoses [23–25]. Current distribution patterns, genetic structure, and the phylogenetic relationships of endemic species in the study area are primarily determined by past climate changes and the Pleistocene glaciation cycles [6, 36, 37]. During glaciation periods, most of the lower- and mid-elevated areas in Central Asia were not covered by ice sheets. They most likely experienced a cooling effect through the albedo from high-altitude glaciers, which advanced beyond their present positions (ca. 600–1000 m lower than today) in the Pamir and Tian Shan mountain regions [38, 39]. Many endemic species survived in the interglacial refugia in deep, wide valleys (e.g. *Ostrowskia magnifica* Regel, *Potentilla kulabensis* Th.Wolf, *Ranunculus chodzhamastonicus* Ovcz. & Junussov) or on steep rock faces in the montane belt, such as *S. regelii* and *S. sewerzowii*. These species adapted to the harsh, but relatively stable conditions in the highest elevations and have thrived in these habitats until now [40].

The distribution of *S. regelii* and *S. sewerzowii* is restricted to the western outcrops of the Central Asian mountains, however, they are characterised by significant differences in distribution patterns (horizontal and vertical geographical disjunction). Our molecular analyses, based on both combined five cpDNA regions as well as DArT SNPs, confirm that they share a common ancestor with species of *Asyneuma* and *C. alberti*, however afterwards, they evolve independently. Our results support also the earlier findings of Xu & Hong [35] that the genus cannot be considered monophyletic. Based on the character of corolla, deeply (until the base) divided into 5 lobes, *S. regelii* was described at first as *Phyteuma regelii* Trautv., being later transferred to *Asyneuma* as *A. regelii* (Trautv.) Bornm. and subsequently to *Podanthum* Boiss.*, P. regelii* (Trautv.) O.Fedtsch. & B.Fedtsch*.* While *S. sewerzowii* was described as *Campanula sewerzowii* Regel [41]. In accordance with the results of our phylogenetic analyses, *S. regelii* exhibits an inconclusive locality within the tree and is grouped either as a sister clade to the *Asyneuma* or *Sergia sewerzowii-Campanula alberti* clades. Whereas *Sergia sewerzowii*, in all analyses except ITS, is grouped with *C. alberti*, to which it exhibits much more morphological similarity (considering the corolla morphology) than to *S. regelii*. The ITS-based phylogenetic tree indicates that both *Sergia* species split from the common ancestor, however, it failed to resolve phylogenetic relationships between *Sergia*, *C. alberti* and *Asyneuma*. Our findings are related to the results of Jones et al. [34], where *C. alberti* is grouped within a common clade with *Asyneuma* and some other taxa from *Petromarula* Vent. ex R.Hedw*.*, *Physoplexis* Schur and *Campanula* L. Whereas, they differ from those of Xu & Hong [35], where *C. alberti* is placed within a common subclade with, e.g., *C. stevenii* M.Bieb*.*, *C. wolgensis* P.A.Smirn*.*, and *C. persicifolia* L. Our results therefore contribute to establishing the taxonomic position of *C. alberti* and confirm that it should not be included to the *C. stevenii* species-group [42]. In our studies, samples of *C. alberti* are grouped together with *S. sewerzowii* in both cpDNA and DArT analyses. These two mentioned above taxa split probably in the late Pliocene-early Pleistocene similarly as the other species of rocky-related endemic plants of this region, i.e., *C. lehmanniana* and *C. eugeniae* [7], and can be also regarded altitudinal vicariants within the Tian Shan Mts. Based on the morphology of the flowers, the corolla of *S. sewerzowii*, that is deeply divided into 5 longer than wide lobes, fused in the lower part (Fig. 1C), is more similar to the corolla of *C. alberti* than to *S. regelii* (which corolla lobes are much narrow and divided into 5, fused only at the base, lobes). The three species are also phylogenetically closely related to *Cylindrocarpa sewerzowii* (Regel) Regel, occurring mainly in southern Kazakhstan [34, 35, 43]. Having in mind that *S. regelii* has an inconsistent position within the *Sergia-Asyneuma* clade, it can not be excluded that during Pleistocene glaciations the range of these two *Sergia* species and *C. alberti* could be much broader, and similarly as in the case of other cold-adapted alpine species [6, 7], gene flow events between these taxa may have occurred in the past. Since all of the above-mentioned species (except *C. alberti*) were grouped by Xu & Hong [35] within one common clade, they suggest merging them (together with selected species from such genera as *Asyneuma*, *Campanula* or *Phyteuma* L., etc.) into the genus *Phyteuma* with the appropriate new circumscription. On the other hand, taking into account that the phylogenetic relationship within Campanulaceae is still unresolved [31–35], it is worth considering a monophyletic approach to the genus *Campanula*, as was recently proposed by Sennikov in Tojibaev et al. [44], who transferred all the Tian-Shanian species of *Adenophora* Fisch., *Asyneuma, Cylindrocarpa, Phyteuma* and *Sergia* to *Campanula*. It is worth mentioning that a similar monophyletic approach was applied also for other genera, such as *Carex* L. or *Prunus* L., with confused phylogenetic relations between their members [45–49].

Additional support for the view of the long and independent evolution of *S. regelii* and *S. sewerzowii* is the significant difference in their realised niches. Both species grow on rocky substrate, but *S. regelii* prefers much higher elevations. Consequently, the habitat of *S. regelii* is characterised by lower temperatures, especially during summer, and lower precipitation. Among the bioclimatic variables, temperature (bio7) mainly influences the distribution of *S. regelii*, whereas precipitation (bio17, bio19) for *S. sewerzowii*. All of these ecological differences are mirrored in the response of the studied endemics to future climate warming.

A remarkable increase in temperature (0.4 °C/decade) and moderate changes in precipitation, with a wetting trend in spring and a drying trend in summer, are expected in future in Central Asia [50]. Although the future forecasts for both studied species are similar, their responses to predicted climate change differ. Up to 64.4% of potential future range loss is expected for *S. regelii*. According to the model, the species will try to escape to higher elevations with cooler climates (statistically significant altitude shift observed for the RPC8.5 model in 2061–2080 with the lower boundary of the distribution moving upward to ca. 700 m). However, upward migration in the mountains is limited because *S. regelii* could reach the limits of its tolerance to such environmental conditions as precipitation, especially in the summer, which could significantly decrease its fitness. Additional stress may occur in the long-term perspective from potential competition with other species escaping from climate warming into new areas at higher elevations [51]. Finally, the species will remain only in small high-mountain refugia, and thus become critically threatened with extinction in the near future. The described consequences, with severe range contractions as a response to recent climate change, are already being observed [52, 53] and/or predicted for many alpine species [54–56], including chasmophytic plant species from the Mountains of Central Asia, such as *Stipa zeravshanica* [6] and *C. lehmanniana* [7].

*Sergia sewerzowii*, which is distributed farther north than *S. regelii* and at lower elevations, shows another possible scenario of alpine species’ response to predicted climate warming that includes a gradual range extension to the north with previous loss of habitats in the south ^6^. The level of expansion is higher when the more pessimistic scenario (RCP8.5) is applied, informing that *S. sewerzowii* possesses high tolerance to warmer temperatures. However, due to the island character of rocky habitats and other intrinsic (restricted dispersal ability) and extrinsic (human activity) factors, the optimistic scenario predicted for *S. sewerzowii* is no guarantee that the species will outlast future climate changes successfully. Although its overall potential habitat area would increase in response to climate change, the species could lose part of its habitat in the Chatkal (NE Uzbekistan), Karzhan-tau, and Ugam Mts (S Kazakhstan). *Sergia regelii*, in turn, will face a high risk of habitat loss in the Kuhitang Mts (E Turkmenistan), Baisun-tau, Sarykya, Chulbair, Tubere-Oland (S Uzbekistan), Nuratau, Aktau (E Uzbekistan), and Kuraminian Mts (N Tajikistan), particularly under the pessimistic scenario in 2061–2080. Moreover, such fast changes in distribution under climatic pressure may lead to the loss of genetic variation and, consequently, to decreased adaptive ability. Thus, it is important to protect these shrinking habitats of *Sergia* species in the Central Asian mountains, where future warming will impose severe stress.

Another important output of the study is the mapping of the areas with highly suitable habitats for *S. regelii* and *S. sewerzowii*, from which they have not been reported yet (W Turkestan range in the case of *S. regelii*, and the Karzhan-tau, Ugam, and Pskemsky ranges for *S. sewerzowii*). Since both *Sergia* species are narrow specialists restricted in their distribution to rocky habitats, additional field studies in the newly described areas of potentially high probability of occurrence will contribute to a better understanding of their environmental preferences and limits, which is important from a conservation point of view and will help to calibrate the model. The model-predicted range shift to the north or higher elevations under climate change can be extrapolated to other unique mountain plant species associated with rocky habitats from the Pamir-Alai and western Tian Shan, part of the Mountains of Central Asia biodiversity hotspot. A whole group of cold-adapted chasmophytic species [18, 23] are vulnerable to potential extinction. Therefore, future conservation activities should be complex and cover not one particular species, but the whole complex of alpine plant communities associated with rocky habitats.

## Conclusions

The present study highlights the relationship between climate change-induced range dynamics, niche distinctiveness, and evolutionary history by considering the example of two chasmophytic endemic plant species, *S. regelii* and *S. sewerzowii*, from the Mountains of Central Asia (a biodiversity hotspot). Genome-wide SNP genotyping, together with analysis of ITS and cpDNA regions showed the polyphyletic nature of the genus *Sergia* and revealed that the taxa constitute a common clade with *Asyneuma* species-group. However, *S. sewerzowii* is more closely related to *Campanula alberti* (a species that has never been included in the genus *Asyneuma* nor in *Sergia*) than *S. regelii*. At the same time, we provide that both *S. regelii* and *S. sewerzowii* have long and independent evolution within different areas of Central Asian mountains. The distinctiveness of species is also reflected in the ranges of occurrence and occupied niches. Also, the future distribution model of studied endemics notably differs: *S. regelii* demonstrates a dramatic loss of habitats with a shift of suitable habitats to higher elevations, while the potential range of *S. sewerzowii* increases and shifts to the north. The character of changes in the predicted distribution depends on the occupied ecological niche and is explained by the fact that *S. regelii* prefers much higher elevations with lower temperatures while *S. sewerzowii* adapted to considerably lower elevations with higher temperatures. The results of presented future distribution modelling can be applied to the whole complex of alpine plant communities associated with rocky habitats of the studied biodiversity hotspot, that are threatened by the ongoing climate changes.

## Methods

### Plant material

Data on the current distribution of *S. regelii* and *S. sewerzowii* are based on (1) phytosociological records and plant specimens collected in Tajikistan during fieldwork in 2008–2019 and deposited in the KRA herbarium (all acronyms following Thiers [57]), (2) herbarium specimens deposited in the LE herbarium, (3) GBIF database and other online sources. Material for the genetic study was collected during previously mentioned fieldwork and from herbarium specimens deposited in the KRA and LE herbaria. The list of samples used in this study is presented in Supplementary Material Table S2. The research complies with relevant institutional, national, and international guidelines and legislation.

### Study area

The study area is located in Central Asia and comprises the Pamir-Alai (NW Tajikistan, SE Uzbekistan, and E Turkmenistan) and western Tian Shan Mountains (S Kazakhstan), characterised by complex climatic conditions due to variable altitudes and orographic effects (Supplementary Material Fig. S6).

The territory of Pamir-Alai belongs to the temperate climatic zone. It is characterised by relatively high insolation, low percentage of cloud cover, high amplitude of annual temperatures, low humidity, and low precipitation. The climate is controlled by two pressure systems: a north-easterly inflow of cold air originating from the Siberian High during winter and a north-westerly inflow of dry air from the Azores High in summer [58]. Therefore, the climate is extremely continental with cold winters, hot and dry summers, and a maximum precipitation in spring. The average annual temperature is 13˚C − 14˚C, the average temperature of January is 0˚C − 2˚C, the average temperature of July is 26˚C − 28˚C, annual precipitation is 400 − 600 mm [59]. In addition, at the highest elevations (> ~ 2500 m a.s.l.), the climate is strongly affected by altitude displaying a more alpine climate regime [58]. In contrast to the lowlands, at alpine elevations, the average temperatures during mid-summer are lower (between 9.7 °C and 13.5 °C) and the annual precipitation is higher [60]. The limit of perpetual snow is at an altitude of 3,500–3,600 m in the western Pamir Alai Mts [61].

The climate of western Tian Shan is typical of the Central Asian mountains, where summer is hot and dry, with air temperatures up to + 30 °C in the mid-mountains and a cold, snowy winter. The average annual precipitation for the mid-mountain zone is about 700 mm, for the high mountain − about 1000 mm. Precipitation is distributed by season as follows: winter − 28%, spring − 38%, summer − 9%, autumn − 25%. Average annual air temperature is + 9.6 °C, the number of frost-free days is 185, average temperature in January is − 3.1 °C, while in July it is + 22.3 °C (Tasaryk meteorological station, 1936–2000, Kazakhstan) [62]. The relief of the western Tian Shan is characterised by very steep slopes in the high- and mid-mountain belts (down to ca. 1400 m) that are replaced by a low-mountain belt with less steep slopes, except for canyons near streams and rivers [63].

### DNA extraction

The genomic DNA was extracted from dried leaf tissues. After grounding plant tissues to a fine powder by using a mixer mill (MM400 Retsch) with 3–5 mm glass beads, genomic DNA was isolated using the Genomic Mini AX Plant kit (A&A Biotechnology, Poland). The purity and concentration of extracted DNA were checked using a NanoDrop ND-1000 spectrophotometer (Thermo Fisher Scientific, USA), whereas the quality was verified by 1% agarose gel electrophoresis. Due to the DArTseq genotyping procedure, the samples were diluted to the concentration of 70 ng/μL, whereas for PCR (ITS and cpDNA) samples were adjusted to 10 ng/μL.

### ITS and cpDNA amplification and sequencing

The nuclear ribosomal Internal Transcribed Spacer region (including ITS1, 5.8S, and ITS2) was amplified using standard ITS5 forward and ITS4 reverse primers [64]. Amplification reactions were performed in a total volume of 25 µl, containing 2.5 μl of 10X DreamTaq Green Buffer (Thermo Scientific, USA), 0.3 μl of 10 mM dNTPs MIX (A&A Biotechnology, Poland), 0.3 μl of 5 U/μl DreamTaq Green DNA Polymerase (Thermo Scientific, USA), 0.5 μl of each primer (10 pmol/µl), 0.1 µl of BSA (20 mg/ml; Thermo Scientific, USA) and 1 μl of template DNA (10 ng/µl). Ultrapure H_2_O was added to obtain a total volume of 25 μl. Amplification of the ITS region was performed in a programmable T100 Thermal Cycler (Bio-Rad, USA) under the following thermal conditions: initial denaturation for 3 min at 94 °C followed by 25 cycles of denaturation for 1 min at 94 °C, annealing for 2 min at 50–56 °C and elongation for 2 min at 72 °C, with final elongation for 7 min at 72 °C.

For cpDNA analysis, we chose five regions used in previously published studies on the family *Campanulaceae* [34, 65, 66], namely *pet*D*, trn*K*-psb*A*, rpl*16*, psb*D*-trn*T*,* and *trn*S*-trnf*M. All regions were amplified using the previously described primers (Supplementary Material Table S5).

PCR conditions in the total volume of 25 μL were: 2.5 μl of 10X DreamTaq Green Buffer (Thermo Scientific, USA), 0.3 μl of 10 mM dNTPs MIX (A&A Biotechnology, Poland), 0.3 μl of 5 U/μl DreamTaq Green DNA Polymerase (Thermo Scientific, USA), 0.2 μl of each primer (10 pmol/µl), 0.2 µl of BSA (20 mg/ml; Thermo Scientific, USA) and 1 μl of template DNA (10 ng/µl). Ultrapure H_2_O was added to obtain a total volume of 25 μl. Amplification of the cpDNA regions was performed in a programmable Eppendorf® Mastercycler. For the *psb*D-*trn*T, *trn*S-*trnf*M, *trn*K*-psb*A, and *rpl*16 regions, we slightly modified the parameters described in Ronikier et al. [66]: initial denaturation for 10 min at 94 °C, followed by 30 cycles that include: 45 s denaturation at 94 °C, 1 min of annealing at 52–60 °C (57–60 °C for *psb*D*-trn*T, 55 °C for rpl16, 58 °C for *trn*S*-trnf*M, 52–55 °C for *trn*K*-psb*A), 2 min of extension at 72 °C, and a final extension of 10 min at 72 °C. For the *pet*D region, we used the following parameters: initial denaturation for 1.5 min at 96 °C, followed by 35 cycles of 30 s at 95 °C, 1 min at 50 °C and 1.5 min at 72 °C, and a final extension of 20 min at 72 °C [67].

In both cases, agarose gel electrophoresis was used to detect the presence or absence of the target sequence and the length of the fragment. Prior to sequencing, PCR products were purified using the Exo-BAP Kit (EURx, Poland). PCR products were sent to an external company (Genomed, Poland) for paired-end Sanger sequencing. The resulting sequences were manually verified and aligned using BioEdit ver. 7.0.5.3 [68].

### Genomic library preparation, DArT sequencing and DArT data filtering

DArTseq is a genome complexity reduction method which implements the sequencing of representations on the Next Generation Sequencing (NGS) platform, optimised for each organism and application in order to select the most appropriate complexity reduction method [69–71]. Therefore, the method has been used successfully in many ecological, evolutionary, population genomic, phylogenetic, and phylogeographic studies [72–77]. Genome complexity reduction using restriction enzymes and high-throughput polymorphism detection [69] was performed by Diversity Arrays Technology Pty Ltd (Canberra, Australia). Based on testing several enzyme combinations for complexity reduction, Diversity Arrays Technology Pty Ltd selected the PstI-MseI method for *Campanula*.

This section was performed according to the previously reported procedures [69]. Briefly, all DNA samples were processed in digestion/ligation reactions as described by Kilian et al. [69], but replacing a single PstI-compatible adaptor with two different adaptors corresponding to two different restriction enzyme overhangs. Only "mixed fragments" (PstI-MseI) were effectively amplified by PCR. After PCR, equimolar amounts of amplification products from each sample of the 96-well microtiter plate were bulked and applied to c-Bot (Illumina, USA) bridge PCR, followed by sequencing on the Hiseq2500 (Illumina, USA). The proprietary DArT analytical pipelines were used to generate sequences from each lane. At the beginning poor-quality sequences (the fastq files) were filter away. As a result, ca. 2.5 mln sequences per barcode/sample were identified and used in the marker call step. For the downstream analyses, we applied co-dominant single nucleotide polymorphisms (SNPs) markers processed in R v. 4.0.3 [78] with the additional package dartR v.2.3.3 [79]. For most of the analysis, we applied the following filtration steps: (1) monomorphic loci were removed, (2) loci identified (= called) in greater than 60%, (3) loci with a scoring reproducibility of 100% were kept, (4) SNPs that shared secondaries were randomly filtered out to keep only one random sequence tag, (5) SNPs were filtered based on the criteria of a minor allele frequency (MAF) threshold of 1%.

### Species delimitation

We conducted a phylogenetic analysis based on ITS and concatenated cpDNA (*pet*D, *trn*K-*psb*A, *rpl*16, *psb*D-*trn*T, and *trn*S-*trn*fM) regions by using Bayesian Inference (BI) analysis performed in MrBayes 3.2.6 [80]. Bayesian Inference (BI) analysis was performed on 25 individuals of representatives of *Sergia*, *Asyneuma*, and *Campanula.* The best-fit substitution model suggested based on the BIC/AIC score calculated in MEGA X [81] was chosen separately for each tree. For the tree based on the concatenated chloroplast regions, we chose the GTR model with a gamma (+ Γ) distributed rates, whereas for ITS data only, we chose K2P model with a gamma (+ Γ) distributed rates. An MCMC simulation was set for 2,000,000 generations, sampling one of every 1000 generations. The first 1000 of iterations were discarded as a burn-in. We also used DArTseq derived SNPs to visualise relationship among studied species (24 individuals and 1047 SNPs) by using Neighbor Joining (NJ) method implemented in dartR package [79]. Based on the results of our studies as well as phylogenetic studies of Jones et al. [33], six species (*C. cervicaria* L.*, C. incanescens* Boiss.*, C. glomerata* L.*, C. rapunculoides* L.*, C. sibirica* L.*, C. trachelium* L.) distantly related to the group of interest, were selected as an outgroup to root the BI and NJ trees. All BI and NJ trees were placed into visual form using FigTree v. 1.4.0 software [82]. A complete list of individuals used in the study and an explanation of abbreviations of samples used in phylogenetic analyses can be found in the Supplementary Material Table S2.

### Data sources and variables selection for ecological niche modelling (ENM)

In order to model the potential distribution of *S. regelii* and *S. sewerzowii*, 34 variables were tested as predictors (Supplementary Material Table S6). In particular, 19 bioclimatic layers, solar radiation data, and elevation were obtained from the WorldClim version 2.1 database [83], the remaining 11 soil variables from a 5–15 cm depth were downloaded from the ISRIC database [84]. The spatial resolution of all layers was 30 arc-seconds (ca. 1 × 1 km). The layers were processed using a system with identical projection (WGS84), cell size, and extent, cropped to the region of interest (Central Asia) and converted into.asc format using the Spatial Analyst and Conversion tools in ArcGIS 10.5 (ArcGIS, 2016). After eliminating redundant presences in each 1 × 1 km grid (spatial resolution of the variables used), 61 localities of *S. regelii* and 19 of *S. sewerzowii* were used for ecological niche modelling. The Pearson correlation coefficient (r) was used to eliminate cross-correlation among variables. Variables with coefficient values less than -0.8 or more than 0.8 were treated as correlated. The selection among correlated variables was based on the variables’ contribution to the model and our own experience and knowledge about the species’ ecology and the region in order to keep ecologically meaningful predictors. This reduction of predictor variables resulted in the inclusion of 10 and 11 variables in the distribution models of *S. regelii* and *S. sewerzowii*, respectively (Table 1).

To predict the future potential distribution of studied species, we used MIROC6 [85], ACCESS-CM2 [86], and HadGEM3-GC31-LL [87] climate change models from CMIP6 (Coupled Model Intercomparison Project Phase 6) that were downloaded from the World Climate Database version 2.1 (30 arc-second spatial resolution) [83]. Representative concentration pathways (RCPs) for minimum (2.6 W/m^2^ of the total radioactive forcing) and maximum (8.5 W/m^2^) emission hypotheses over the periods 2041–2060 and 2061–2080 were selected for further projections. The RCPs reflect potential radiative forcing by 2100 compared with the pre-industrial values of + 2.6 W/m^2^, which is optimistic, or + 8.5 W/m^2^, which is more pessimistic and reflects high emission levels of greenhouse gases [88].

### Ecological modelling

The MaxEnt program used in this study was downloaded from biodiversityinformatics.amnh.org/open_source/maxent/ (free of charge for scientific research activities). Among the basic settings, random seed was selected, 25% of occurrence records were used to test the model and 75% for training, replicated run type is a subsample, the number of replicates is equal to 100. Among the advanced settings, a 10-percentile training presence threshold rule was applied [89]. The remaining settings were kept as default. The Maxent results were validated using the threshold-independent area under the curve (AUC) of receiver operating characteristics (ROC). Jackknife analyses were performed to assess the importance of the variables [90]. The results of the Maxent modelling in the form of potential species distribution maps ranged in value from 0 to 1, according to the probability of species occurrence in each particular area (cell on map). To produce presence/absence maps of *S. regelii* and *S. sewerzowii* potential distribution, the Equal test sensitivity and specificity logistic threshold were selected among the different threshold values produced by Maxent as the most ecologically meaningful for the studied species. The threshold equals 0.1313 for *S. regelii* and 0.2263 for *S. sewerzowii*. The reclassify tool in ArcGIS 10.5 was used to create binary maps (suitable or unsuitable areas). To produce a consensus map of potential future distribution based on the three climate models (MIROC6, ACCESS-CM2, and HadGEM3-GC31-LL), the Cell Statistics tool in ArcGIS 10.5 with a majority voting approach was applied [91]. To assess the changes in the distribution of studied species during the twenty-first century, current and future presence/absence maps were compared. The changes in area were classified as (1) gain, (2) loss, and (3) unchanged [92].

### Niche model analyses

To compare the niches of *S. regelii* and *S. sewerzowii*, 25000 random background points were extracted from a buffer zone of 20 km around known localities of occurrence, using ArcMap 10.5. Then, the values selected for the modelling variables (except elev, srh, and snd) were extracted for known localities of the studied species and for the random background points. The overlap between realised niches of *S. regelii* and *S. sewerzowii* was calculated using Schoener’s D index and I statistic, ranging from zero (no overlap) to one (complete overlap). Niche equivalency and niche similarity tests were applied to test the hypotheses of niche conservatism and niche divergence [93]. All calculations were performed using the ecospat package v 3.2 [94] in R [78]. The niche equivalency and similarity hypothesis was tested by comparing the observed niche overlap to overlaps between random niches, built from random reallocations of occurrences. The process was repeated 1000 times to generate a null distribution of niche overlap values of simulated randomly permuted niches. For both the niche equivalency and similarity tests, the argument = “greater” (overlap greater than expected by chance) to test the conservatism hypothesis, and the argument = “lower” (overlap lower than expected by chance) to test the divergence hypothesis were used [93, 94]. In the case of the niche equivalency test, the null hypothesis of niche equivalency was accepted if the observed value of D/I fell within a density of 95% of the simulated values, whereas in the case of the niche similarity test, the null hypothesis was accepted when observed overlap values were greater than 95% of the simulated values. Principal Component Analysis (PCA) was performed to visualise niche overlap. The PCA scores of the two compared niches were projected onto a grid of cells bounded by minimum and maximum PCA scores in the study areas. Additionally, niche dynamics along the gradients of all variables selected for the analysis were visualised [94].

### Supplementary Information

**Supplementary Material 1.**

## Data Availability

The SNP dataset derived from the DArTseq pipeline in the genlight format as well as alignments from ITS and cpDNA in the fasta format are available via Figshare repository, https://doi.org/10.6084/m9.figshare.21707294. The ITS sequences used for comparative analyses and phylogenetic trees reconstruction have been deposited in GenBank database (Accession Numbers: OP998105-OP998118).
