# Supplementary material for: Insight into the phylogeny and responses of species from the genus Sergia (Campanulaceae) to the climate changes predicted for the Mountains of Central Asia (a world biodiversity hotspot)
Source: BMC Plant Biol. 2024 Apr 1;24:228. doi: 10.1186/s12870-024-04938-4 (PMC10986085; doi:10.1186/s12870-024-04938-4)
Supplement: Supplementary file 1 — Supplementary Material 1. [file 12870_2024_4938_MOESM1_ESM.docx]

**Insight into phylogeny and responses of species from the genus *Sergia* (Campanulaceae) to the climate changes predicted for the Mountains of Central Asia (a world biodiversity hotspot)**

Lizaveta Vintsek^1*^, Ewelina Klichowska^1^, Arkadiusz Nowak^2, 3^, Marcin Nobis^1*^

^1^Institute of Botany, Faculty of Biology, Jagiellonian University, Gronostajowa 3, 30–387 Kraków, Poland

^2^Polish Academy of Sciences Botanical Garden – Center for Biological Diversity Conservation in Powsin, Prawdziwka 2, 02-973 Warszawa, Poland

^3^Botanical Garden of the Wrocław University, Sienkiewicza 23, 50-335 Wrocław, Poland

*corresponding authors: m.nobis@uj.edu.pl; lizaveta.vintsek@doctoral.uj.edu.pl


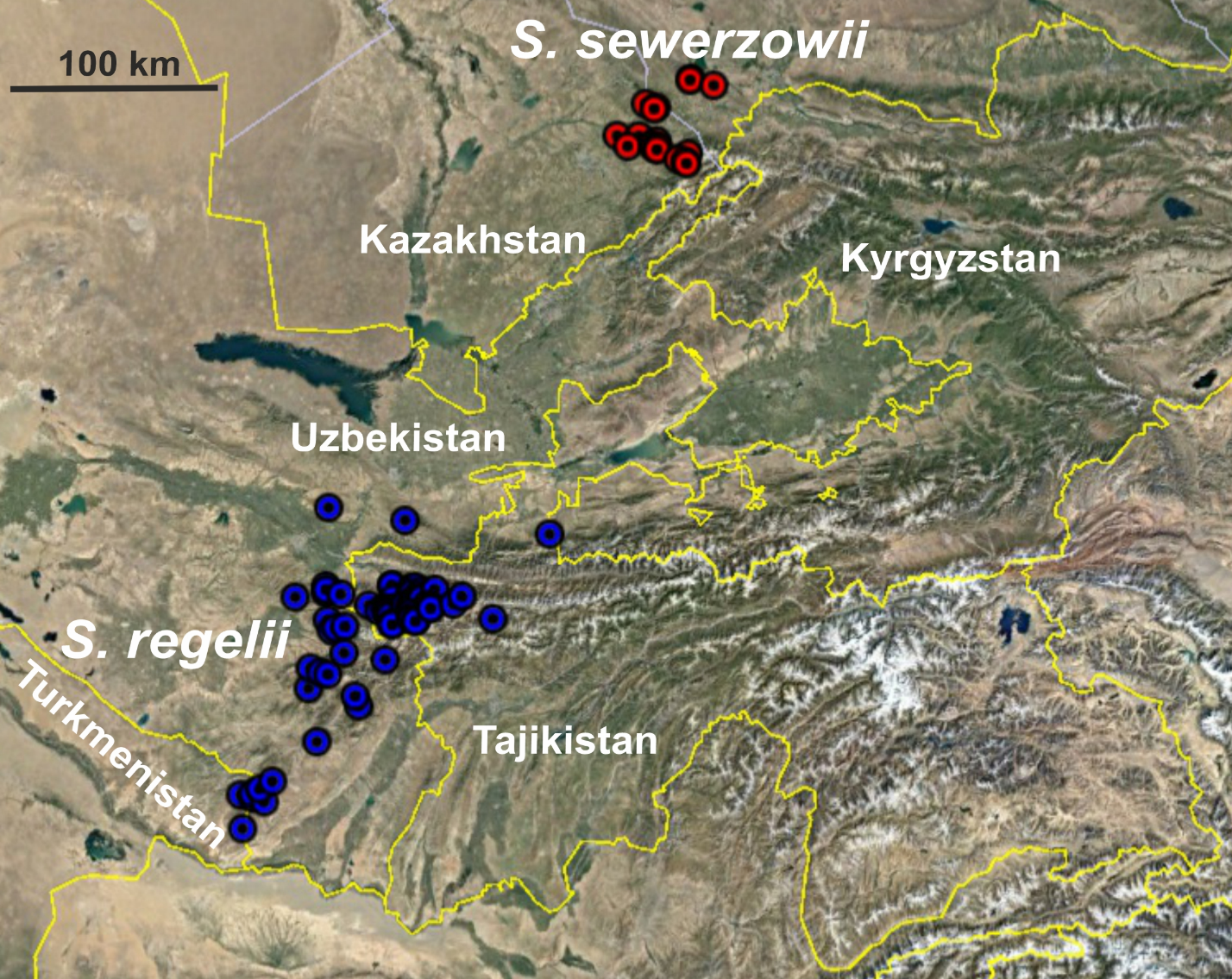


**Supplementary Figure S1.** The distribution of *Sergia regelii* (blue points) and *S. sewerzowii* (red points) in the mountains of Central Asia.


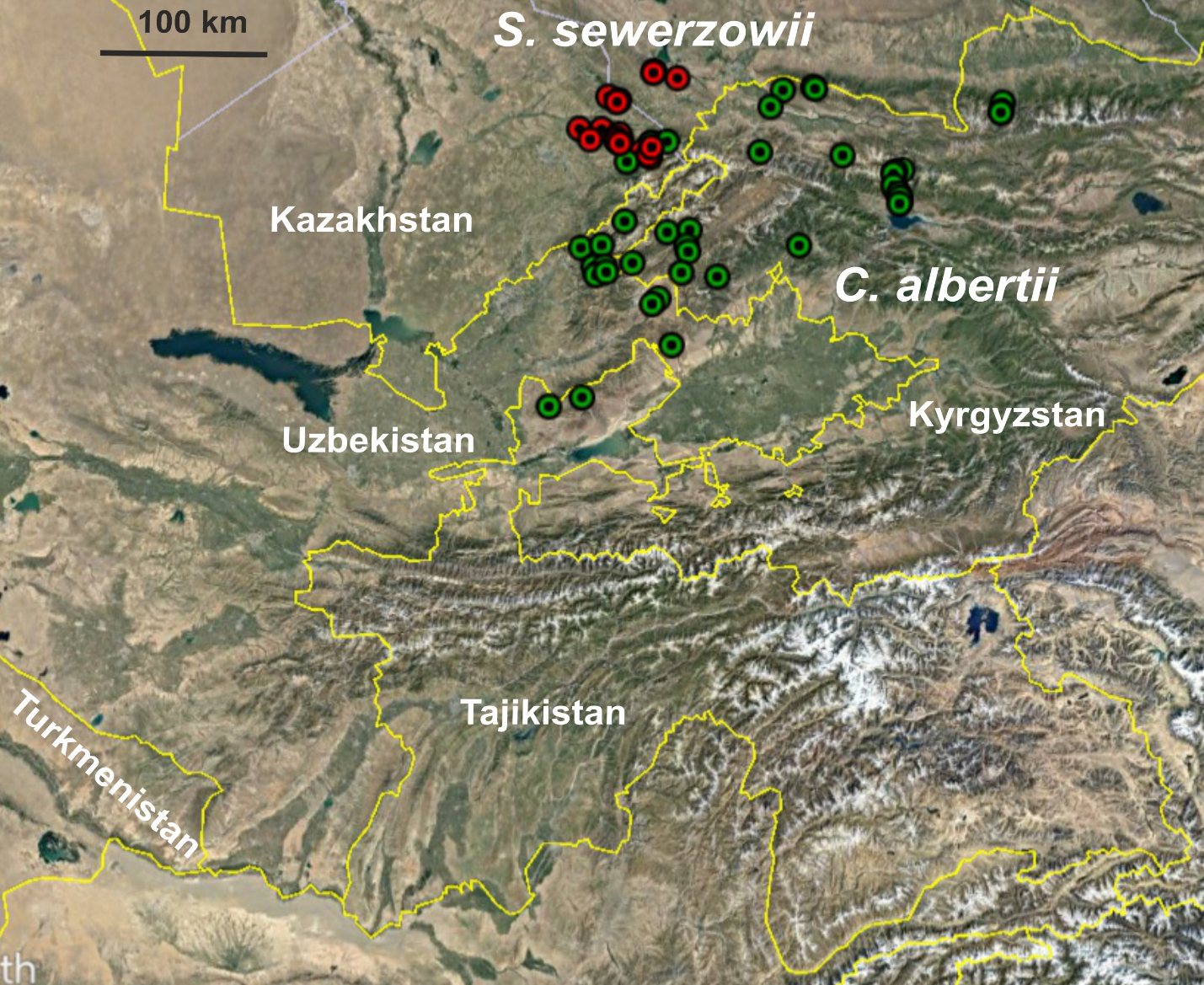


**Supplementary Figure S2.** The distribution of *S. sewerzowii* (red points) and *C. alberti* (green points) in the mountains of Central Asia.


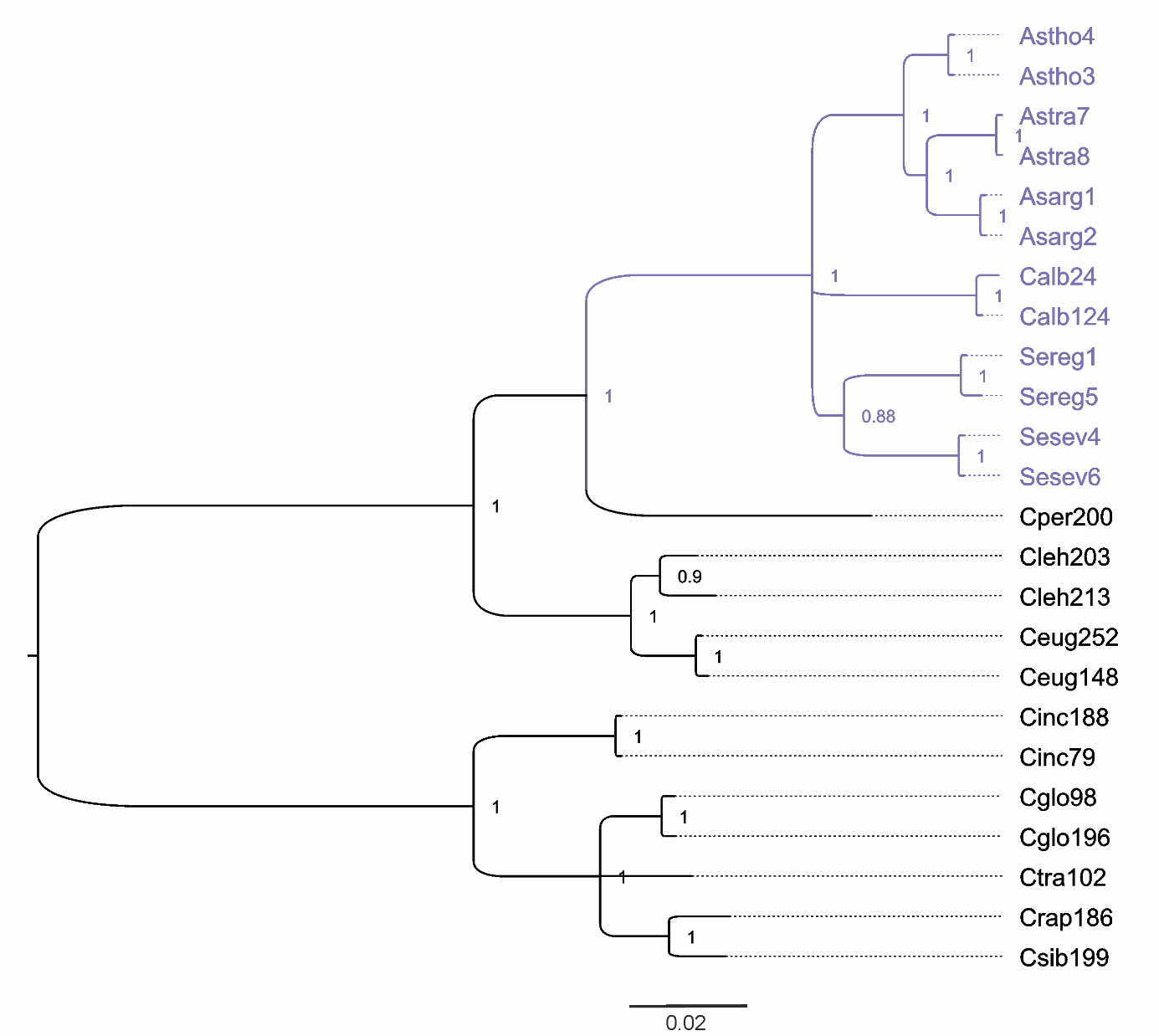


**Supplementary Figure S3.** Bayesian Inference of phylogeny based on ITS region (24 individuals, 688 bp); numbers at the nodes indicates the Bayesian posterior probability values.


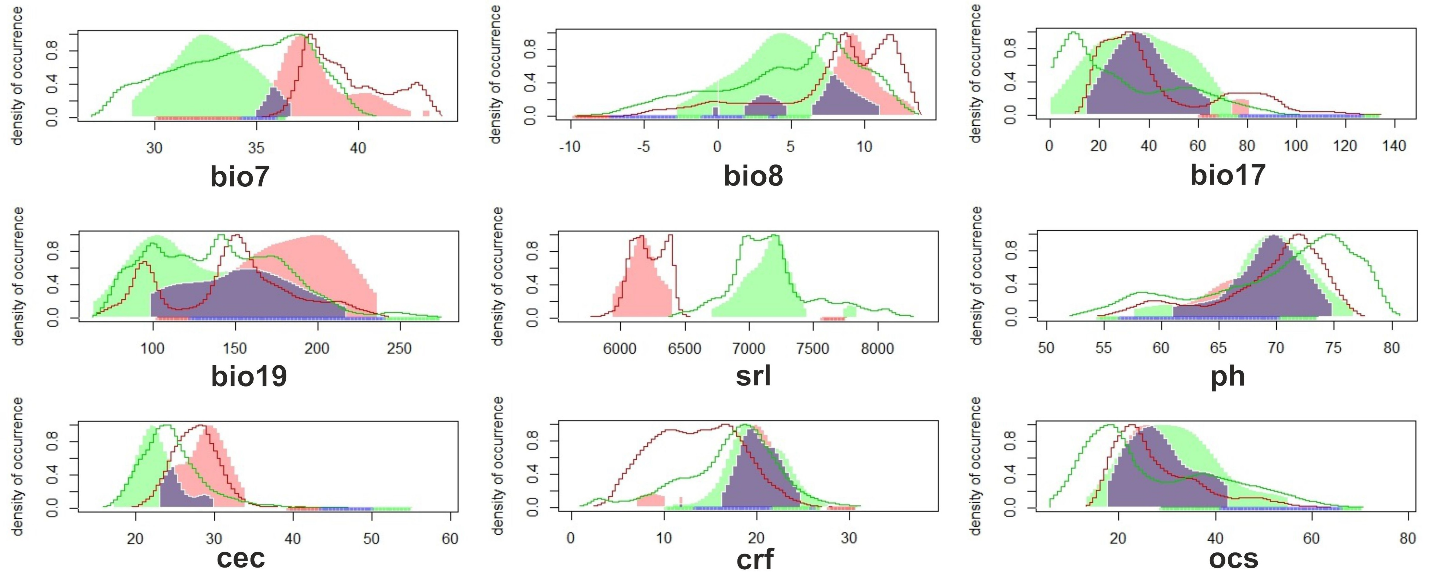


**Supplementary Figure S4.** Visualization of the niche categories (environmental variables) and species density (green shading - environmental space of *S. regelii*, red shading - environmental space of *S. sewerzowii*, blue shading – overlap of environmental spaces). The codes for environmental variables as in Table 1.


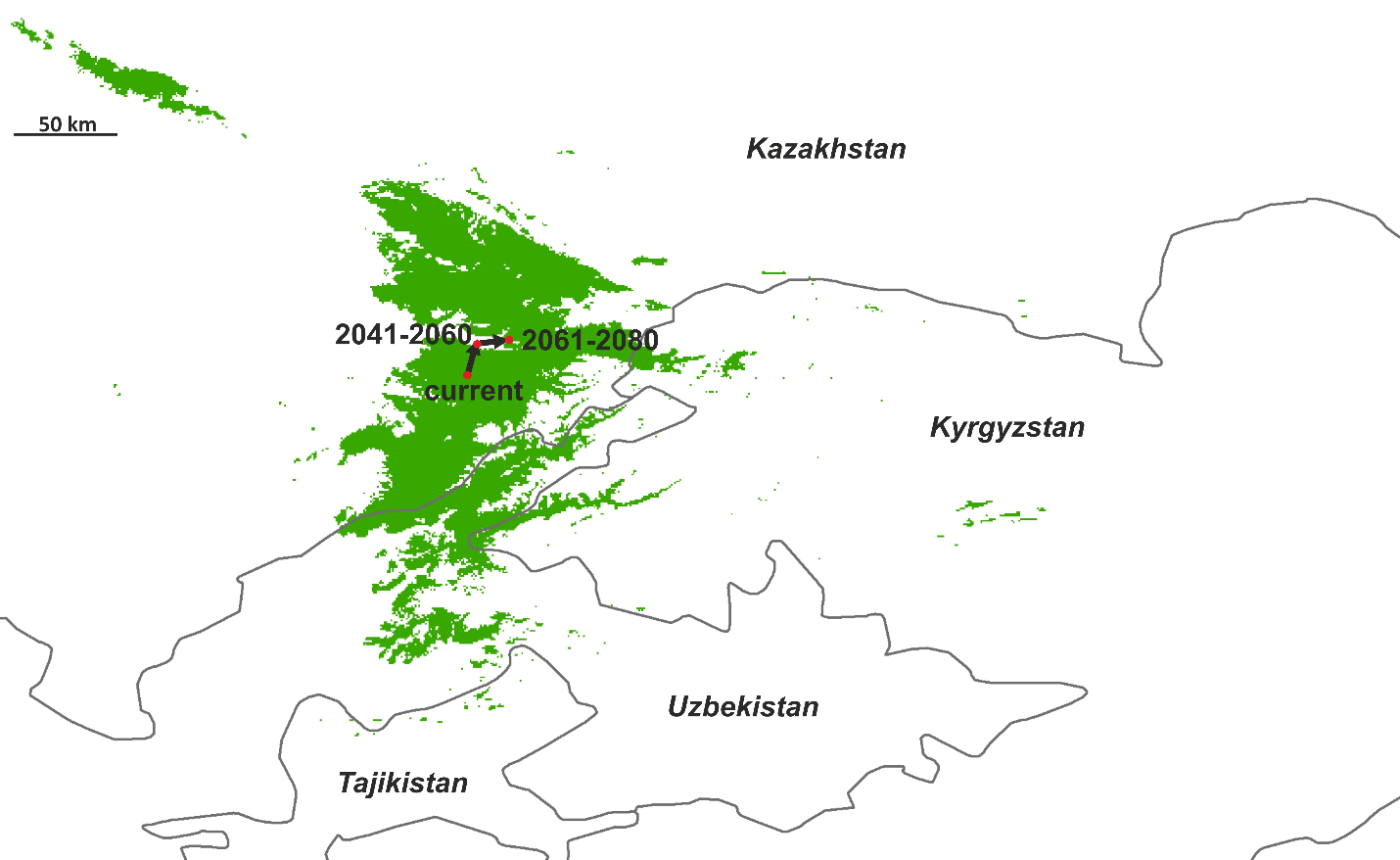


**Supplementary Figure S5.** Changes in centroids of the potential distribution for *S. sewerzowii* from the present time till the future under the RPC8.5 climate scenario. Green shading is the current potential range of *S. sewerzowii*.


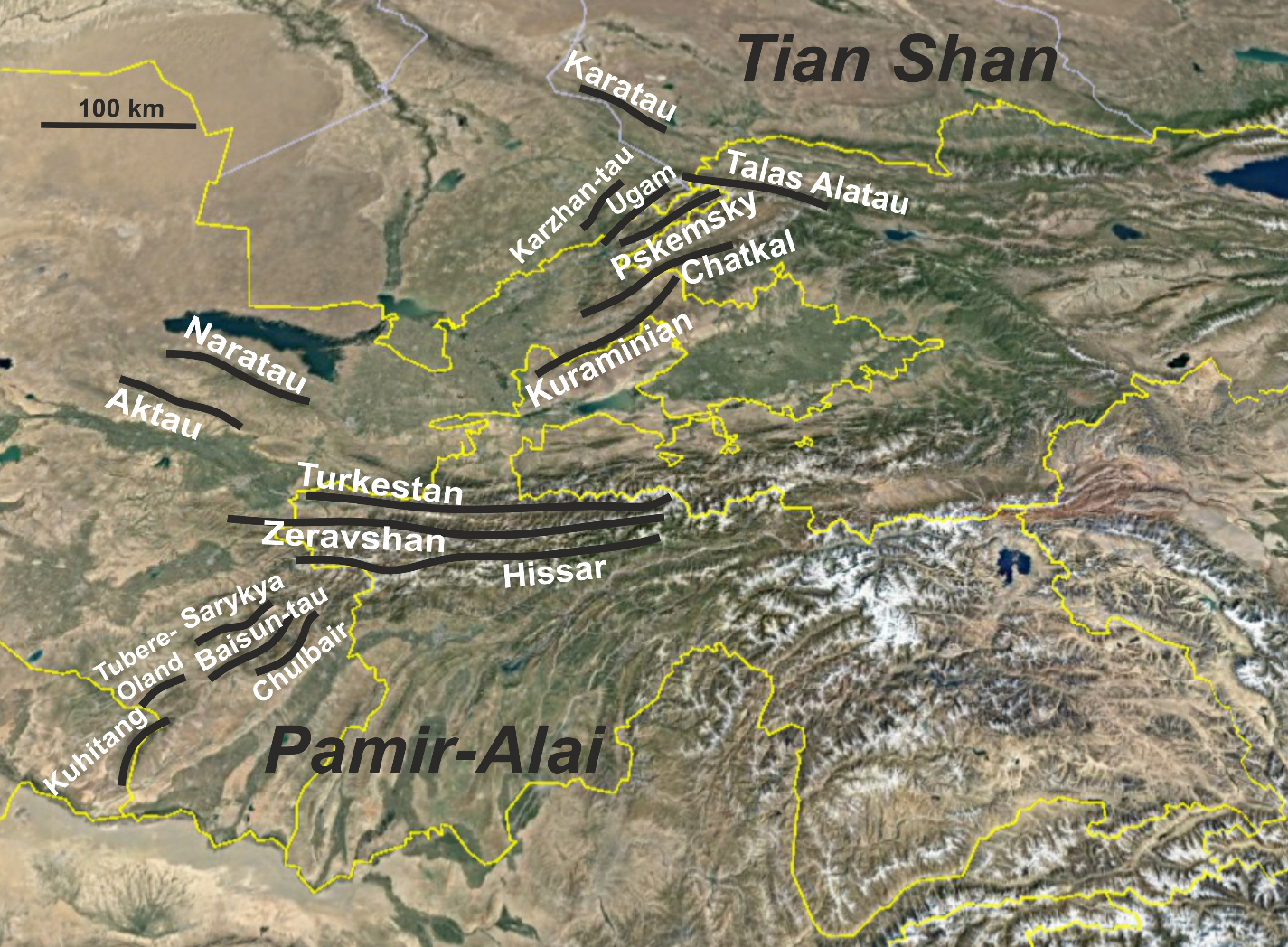


**Supplementary Figure S6.** The map of the study area with the names of the selected mountain ranges in the Pamir-Alai and Tian Shan Mts.

**Supplementary Table S1.** A comparison of the main morphological characters of *S. regelii* and *S. sewerzowii* based on the specimens deposited in LE herbarium (acronym following Thiers ^1^) and literature data ^2,3^.

| Character | S. regelii | S. sewerzowii |
| --- | --- | --- |
| Corolla length (mm) | (5–)7–8.2(–10) | (5–)6.7–7.5(–8) |
| Petals length (mm) | (5–)7–8(–10) | (3–)4.4–6.4(–7) |
| Calyx length (mm) | (1.5–)2.5–6(–7) | (1.5–)2–3(–3.5) |
| Sepals length (mm) | (2.8–)4.5–6(–7.4) | (4–)4.4–6(–7) |
| Stem length (cm) | (10–)12–23(–30) | (5.5–)10–15(–17) |
| Stem leaves length (mm) | (14–)20–30(–35) | (18.5–)20–40(–46) |
| Character of corolla, petals, calyx, sepals, stem, and leaves surface | shortly hairy | glabrous |

**Supplementary Table S2.** List of the Campanulaceae specimens used in the study. The specimens used for the modelling or for the genetic study are marked by plus (“+”) sign. Collectors: M. N. – Marcin Nobis, E. K. – Ewelina Klichowska, A. N. – Arkadiusz Nowak, A. W. – Anna Wróbel.

| Species name | Country | Localisation | Habitat | Latitude, N | Longitude, E | Date | Altitude | Exposition | Slope, ° | Collector | Herbarium code | Nr of herbarium sheet | Modelling | Genetic | Genetic sample code | Link |
| --- | --- | --- | --- | --- | --- | --- | --- | --- | --- | --- | --- | --- | --- | --- | --- | --- |
| *Asyneuma argutum* | Tajikistan | Fann Mts, forest near Arch river valley, NW of Saryrag settl. | birch forest near the river | 39.05333 | 68.31944 | 16.06.2008 | 2410 |  |  | M. N. | KRA | 456177 |  | + | Asarg1 |  |
| *Asyneuma argutum* | Tajikistan | Hissar Mts, left side of the Takob valley, Sari Szara mountain slope | rocky slope |  |  | 05.06.2007 | 1850 | N |  | M. Kozak, M. N., A. N. | KRA | 455537 |  | + | Asarg2 |  |
| *Asyneuma thomsoni* | Kyrgyzstan | ca. 22.5 km NW from Toktogul |  | 42.059527 | 72.8213055 | 28.06.2017 |  |  |  | M. N., E. K., A. W., A. N. | KRA | 476524 |  | + | Astho3 |  |
| *Asyneuma thomsoni* | Kyrgyzstan | W Tien-Shan, Talas Ala-Too Range, Talas Region, ca. 70 km to the SE from the Talas city, near road M41 | shrubs | 42.059916 | 72.82 | 28.06.2017 | 1535 | NE | 30 | M. N., E. K., A. W., A. N. | KRA | 474789 |  | + | Astho4 |  |
| *Asyneuma trautvetteri* | Tajikistan | Fann Mts, W of Kulikalon Lake | high mountain grassland |  |  | 25.06.2008 |  |  |  | M. Kozak, M. N. | KRA | 455542 |  | + | Astra7 |  |
| *Asyneuma trautvetteri* | Tajikistan | Fann Mts, W of Kulikalon Lake | high mountain grassland |  |  | 25.06.2008 |  |  |  | M. Kozak, M. N. | KRA | 455542 |  | + | Astra8 |  |
| *Asyneuma trautvetteri* | Tajikistan | Zeravshan Mts |  |  |  | 2019 |  |  |  | A. N., et al. | KRA |  |  | + | Astra2019/2 |  |
| *Campanula alberti* | Kyrgyzstan | Jalalabad Oblast, Tian-Shan, Tschatkalgebirge, 55 km W of Ala Buka und 5 km SSE of Tschaptschyma-Pass |  | 41.49981 | 70.84103 | 20.07.2015 | 2330 |  |  | G. A. Lazkov | GJO |  |  |  |  | https://www.gbif.org/occurrence/1935668565 |
| *Campanula alberti* | Tajikistan | SW Tian-Szan, roadside near the Karamazorsoi village, Kuramin Mts. | rocks | 40.65667 | 69.92278 | 05.06.2010 | 1540 | S | 0-80ᵒ | M. N. | KRA |  |  |  |  |  |
| *Campanula alberti* | Tajikistan | SW Tian-Szan, roadside near the Karamazorsoi village, Kuramin Mts. | rocks | 40.65667 | 69.92278 | 05.06.2010 | 1540 | S | 0-80ᵒ | M. N. | KRA |  |  |  |  |  |
| *Campanula alberti* | Tajikistan | SW Tian-Szan, roadside near the Karamazorsoi village, Kuramin Mts. | rocks | 40.65667 | 69.92278 | 05.06.2010 | 1540 | S | 0-80ᵒ | M. N. | KRA |  |  |  |  |  |
| *Campanula alberti* | Kyrgyzstan | Talas region, Chychkan River Valley, ca. 17 km to N from Toktogul Reservoir, near road M41 | rocks | 42.00294 | 72.86326 | 04.06.2013 | 1214 |  |  | M. N. | KRA | 485753 |  |  |  |  |
| *Campanula alberti* | Kyrgyzstan | W Tian-Shan, Talas Ala-Too Range, Talas Region, ca. 75 km to the SE from the Talas city, near the road M41 | - | 42.02866 | 72.84018 | 28.06.2017 | 1372 | NE | 15° | M. N., E. K., A. W., A. N. | KRA | 477975 |  |  |  |  |
| *Campanula alberti* | Kyrgyzstan | W Tian-Shan, Talas Ala-Too Range, Talas Region, ca. 75 km to the SE from the Talas city, near the road M41 |  | 42.02866 | 72.84018 | 28.06.2017 | 1372 | NE | 15° | M. N., E. K., A. W., A. N. | KRA | 477976 |  |  |  |  |
| *Campanula alberti* | Kyrgyzstan | ca. 22.5 km NW from Toktogul |  | 42.05946 | 72.81889 | 28.06.2017 | 1546 |  |  | M. N., E. K., A. W., A. N. | KRA | 476226 |  |  |  |  |
| *Campanula alberti* | Kyrgyzstan | ca. 22.5 km NW from Toktogul |  | 42.05946 | 72.81889 | 28.06.2017 | 1546 |  |  | M. N., E. K., A. W., A. N. | KRA | 476525 |  |  |  |  |
| *Campanula alberti* | Kyrgyzstan | ca. 32 km SW from Taldy Bulak and ca. 31 km NNW from Toktogul |  | 42.14018 | 72.82098 | 28.06.2017 | 1781 |  |  | M. N., E. K., A. W., A. N. | KRA | 476547 |  |  |  |  |
| *Campanula alberti* | Kyrgyzstan | ca. 32 km SW from Taldy Bulak and ca. 31 km NNW from Toktogul |  | 42.14018 | 72.82098 | 28.06.2017 | 1781 |  |  | M. N., E. K., A. W., A. N. | KRA | 476506 |  |  |  |  |
| *Campanula alberti* | Kyrgyzstan | ca. 32 km SW from Taldy Bulak and ca. 31 km NNW from Toktogul |  | 42.14018 | 72.82098 | 28.06.2017 | 1781 |  |  | M. N., E. K., A. W., A. N. | KRA | 476504 |  |  |  |  |
| *Campanula alberti* | Kyrgyzstan | ca. 32 km SW from Taldy Bulak and ca. 31 km NNW from Toktogul |  | 42.14018 | 72.82098 | 28.06.2017 | 1781 |  |  | M. N., E. K., A. W., A. N. | KRA | 476505 |  |  |  |  |
| *Campanula alberti* | Kyrgyzstan | ca. 32 km SW from Taldy Bulak and ca. 31 km NNW from Toktogul |  | 42.14018 | 72.82098 | 28.06.2017 | 1781 |  |  | M. N., E. K., A. W., A. N. | KRA | 476546 |  | + | Calb124 |  |
| *Campanula alberti* | Kyrgyzstan | Central Tian-Shan ca. 32 km NNW of Toktogul, ca. 92 km W of Suusamyr | rocks | 42.15687 | 72.84111 | 30.06.2015 | 1874 |  |  | M. N., A. N. | KRA | 500719 |  |  |  |  |
| *Campanula alberti* | Kyrgyzstan | W Tian-Shan, Talas Ala-Too Range, Talas Region, ca. 65 km to the SE from the Talas city, near road M41 |  | 42.16430 | 72.90749 | 28.06.2017 | 2048 | SE | 30-40° | M. N., E. K., A. W., A. N. | KRA | 476516 |  |  |  |  |
| *Campanula alberti* | Kyrgyzstan | W Tian-Shan, Talas Ala-Too Range, Talas Region, ca. 65 km to the SE from the Talas city, near road M41 |  | 42.16430 | 72.90749 | 28.06.2017 | 2048 | SE | 30-40° | M. N., E. K., A. W., A. N. | KRA | 476515 |  |  |  |  |
| *Campanula alberti* | Kyrgyzstan | W Tian-Shan, Talas Ala-Too Range, Talas Region, ca. 65 km to the SE from the Talas city, near road M41 |  | 42.16430 | 72.90749 | 28.06.2017 | 2048 | SE | 30-40° | M. N., E. K., A. W., A. N. | KRA | 476514 |  |  |  |  |
| *Campanula alberti* | Kyrgyzstan | ca. 15 km S from Sosnovka | rocks | 42.53443 | 73.85842 | 28.06.2017 | 1614 |  |  | M. N., E. K., A. W., A. N. | KRA | 474775 |  |  |  |  |
| *Campanula alberti* | Kyrgyzstan | ca. 15 km S from Sosnovka | rocks | 42.53443 | 73.85842 | 28.06.2017 | 1614 |  |  | M. N., E. K., A. W., A. N. | KRA | 474774 |  |  |  |  |
| *Campanula alberti* | Kyrgyzstan | W Tian-Shan, Kyrgyz Mts., S of Sosnovka | rocks | 42.54611 | 73.86306 | 29.06.2015 | 1480 | W | 30° | M. N., A. N. | KRA | 475102 |  |  |  |  |
| *Campanula alberti* | Kyrgyzstan | W Tian-Shan, Kyrgyz Mts, ca. 20 km S of Sosnovka | rocks | 42.54611 | 73.86306 | 29.06.2015 | 2300 | SW | 60° | M. N., A. N. | KRA | 475127 |  |  |  |  |
| *Campanula alberti* | Kyrgyzstan | W Tian-Shan, Kyrgyz Mts, ca. 20 km S of Sosnovka | rocks | 42.54611 | 73.86306 | 29.06.2015 | 1480 | W | 30° | M. N., A. N. | KRA | 475103 |  |  |  |  |
| *Campanula alberti* | Kyrgyzstan | W Tian-Shan, Chui Region, ca. 66 km to the SW from the city of Biszkek, 6 km to the SW from the Sosnovka village, near the road M41 |  | 42.60549 | 73.88306 | 28.06.2017 | 1322 | SE | 30° | M. N., E. K., A. W., A. N. | KRA | 478456 |  |  |  |  |
| *Campanula alberti* | Kyrgyzstan | W Tian-Shan, Chui Region, ca. 66 km to the SW from the city of Biszkek, 6 km to the SW from the Sosnovka village, near the road M41 |  | 42.60549 | 73.88306 | 28.06.2017 | 1322 |  |  | M. N., E. K., A. W., A. N. | KRA | 474786 |  |  |  |  |
| *Campanula alberti* | Kyrgyzstan | W Tian-Shan, Chui Region, ca. 66 km to the SW from the city of Biszkek, 6 km to the SW from the Sosnovka village, near the road M41 |  | 42.60549 | 73.88306 | 28.06.2017 | 1322 | SE | 30° | M. N., E. K., A. W., A. N. | KRA | 474773 |  |  |  |  |
| *Campanula alberti* | Kyrgyzstan | W Tien-Shan, Talas Ala-Too Range, Talas Region, ca. 65 km to the SE from the Talas city, near road M41 | 862 | 42.1655 | 72.9098888 | 28.06.2017 |  |  |  | M. N., E. K., A. W., A. N. | KRA (gel) |  |  | + | Calb24 |  |
| *Campanula alberti* | Uzbekistan | Samarkand district, Hodjentskiy province, Chomg-ata pass |  | 40.28333 | 69.61667 | 27.05.1914 |  |  |  | O. von Knorring | LE | 269 |  |  |  |  |
| *Campanula alberti* | Tajikistan | Kuramin Range, Altyn-topkan Mts., between Altyn-Topkan and Taboshar cities | juniperus forest | 40.59674 | 69.62271 | 13.07.1962 |  |  |  | V. P. Bochancev | LE | 16 |  |  |  |  |
| *Campanula alberti* | Uzbekistan | W Alatau |  | 41.00000 | 69.00000 | 08.1876 |  |  |  | Regel | LE |  |  |  |  |  |
| *Campanula alberti* | Uzbekistan | Tashkent district, Alatau |  | 41.00000 | 69.00000 | 20.05.1880 |  |  |  | L. Mussa | LE |  |  |  |  |  |
| *Campanula alberti* | Uzbekistan | Chatkal-Akbulak River bassin, meadle part of Koguman-sai | rocks in the juniperus belt | 41.45000 | 70.20000 | 24.07.1972 |  |  |  | R. Kamelin | LE | 307 |  |  |  |  |
| *Campanula alberti* | Uzbekistan | Chatkal-Aktulak River bassin, meadle part of Koguman-sai | rocks in the juniperus belt | 41.45000 | 70.20000 | 24.07.1972 |  |  |  | R. Kamelin | LE | 309 |  |  |  |  |
| *Campanula alberti* | Uzbekistan | S hill of Bolshoy Chimgan Mt. | rock cracks | 41.48333 | 70.05000 | 25.06.1959 |  |  |  | V. P. Bochancev | LE | 551 |  |  |  |  |
| *Campanula alberti* | Uzbekistan | Bolshoy Chimgan, rise to the camp site, rocks up to juniperus tree along Chimgan valley | rocks | 41.48333 | 70.05000 | 20.07.1972 |  |  |  | R. Kamelin | LE | 239 |  |  |  |  |
| *Campanula alberti* | Uzbekistan | W Tian-Shan, Bolshoy Chimgan |  | 41.48333 | 70.05000 | 08.08.1897 |  |  |  | O. A. Fedchenko, B. A. Fedchenko | LE |  |  |  |  |  |
| *Campanula alberti* | Uzbekistan | Chimgan, alpine belt of Bolshoy Chimgan | rock cracks | 41.49444 | 70.05778 | 22.08.1924 |  |  |  | P. Gomolicky | LE |  |  |  |  |  |
| *Campanula alberti* | Kyrgyzstan | Alexandrovsky Range, Kenkil River, Chazhe River valley | juniperus zone, hill | 41.50000 | 73.01667 | 20.07.1930 |  |  |  | M. M. Il'in | LE | 233 |  |  |  |  |
| *Campanula alberti* | Uzbekistan | Chimgan Mts., upper part of of Chimganka River | rocks | 41.55000 | 70.00000 | 29.06.1974 |  |  |  | R. Kamelin | LE | 1413 |  |  |  |  |
| *Campanula alberti* | Uzbekistan | Namangan district, Aflatun River valley (on the pass) |  | 41.66739 | 71.92558 | 26.06.1912 |  |  |  | O. Knorring | LE | 356 |  |  |  |  |
| *Campanula alberti* | Uzbekistan | Namangan district, Aflatun River valley (on the pass) |  | 41.66739 | 71.92558 | 26.06.1912 |  |  |  | O. Knorring | LE | 356 |  |  |  |  |
| *Campanula alberti* | Uzbekistan | Namangan district, Aflatun River valley (on the pass) |  | 41.66739 | 71.92558 | 26.06.1912 |  |  |  | O. Knorring | LE | 356 |  |  |  |  |
| *Campanula alberti* | Uzbekistan | Pskem River valley, meadle part | rocks in the juniperus belt | 41.83333 | 70.30000 | 22.07.1972 |  |  |  | R. Kamelin | LE | 257 |  |  |  |  |
| *Campanula alberti* | Uzbekistan | Syr-Dar district, between Pskem and Nanai |  | 41.86250 | 70.32639 | 01.06.1903 |  |  |  | V. I. Lipsky | LE | 2929 |  |  |  |  |
| *Campanula alberti* | Kyrgyzstan | Uzun-Ahmat, S hill of Susa-Myrsky Range |  | 41.91667 | 72.58333 | 28.07.1881 |  |  |  | A. Fedshov | LE |  |  |  |  |  |
| *Campanula alberti* | Kyrgyzstan | Chatkal |  | 42.10000 | 71.68333 | 1876 |  |  |  | Regel | LE | 2206 |  |  |  |  |
| *Campanula alberti* | Kyrgyzstan | Chatkal |  | 42.10000 | 71.68333 | 1876 |  |  |  | Regel | LE | 2206 |  |  |  |  |
| *Campanula alberti* | Kyrgyzstan | Talas Alatau, Karabura pass. |  | 42.20000 | 71.56667 | 1876 |  |  |  | Regel | LE |  |  |  |  |  |
| *Campanula alberti* | Kyrgyzstan | Karabura |  | 42.20000 | 71.56667 | 1876 |  |  |  | Regel | LE | 2209 |  |  |  |  |
| *Campanula alberti* | Kyrgyzstan | Talas Ala-Too Range, meadle part of Beshtash valley | rocks | 42.27996 | 72.35788 | 01.07.1973 |  |  |  | R. Kamelin | LE | 74-Б |  |  |  |  |
| *Campanula alberti* | Kazakhstan | W part of Talas Ala-Too Range, Aksu-Djabagly Reserve |  | 42.40000 | 71.56667 | 14.07.1956 |  |  |  | Y. D. Soskov | LE |  |  |  |  |  |
| *Campanula alberti* | Kazakhstan | Talas Ala-Too Range, Aksu-Djabagly Reserve, Kshi-Kaindy River valley, below waterfall | sandy rocks | 42.40167 | 70.58250 | 09.07.1950 |  |  |  | I. A. Linchevsky, O. I. Rozhkova | LE | 54 |  |  |  |  |
| *Campanula alberti* | Kazakhstan | NW part of Kirgiz Range, close to Djambula Mt. (Jambyl Mts.), meadle part of Siugaty River | rocky hills, in the cracks | 44.80333 | 73.01667 | 08.06.1963 |  |  |  | V. Goloskokov | LE |  |  |  |  |  |
| *Campanula alberti* | Kyrgyzstan | Central Asia, Tian-shan, Mount Chatkalski Khrebet, Mount Chimgan, in clivis situated northward |  | 41.55000 | 70.00000 | 17.07.1973 |  |  |  | V. Vasák | MA |  |  |  |  | https://www.gbif.org/occurrence/1936565578 |
| *Campanula alberti* | Kyrgyzstan | Toktokul'sky region, left bench of the Chichkan river, cliffs |  | 41.97361 | 72.87528 | 07.06.1977 |  |  |  | R. Aydarova | MO |  |  |  |  | https://www.gbif.org/occurrence/1258595660 |
| *Campanula alberti* | Kyrgyzstan | Leninopol'sky region, Ykele-tay mountain vicinity of the Beysheke village, southern slope |  | 42.63556 | 71.70361 | 22.05.1990 |  |  |  | Sheremetova | MO |  |  |  |  | https://www.gbif.org/occurrence/1258595704 |
| *Campanula alberti* | Kyrgyzstan | Toktogul'sky range, Sysamyrsky range, right bank of the Tonus river, on cliffs |  |  |  | 10.06.1977 |  |  |  | R. Aydarova | MO |  |  |  |  | https://www.gbif.org/occurrence/1258595746 |
| *Campanula alberti* | Tajikistan | Hisar range, Maihura river |  | 39.05000 | 68.71667 | 22.07.1984 |  |  |  | U. Baranova | MW |  |  |  |  | https://plant.depo.msu.ru/public/scan.jpg?pcode=MW0878028 |
| *Campanula alberti* | Uzbekistan | S slopes of Kuraminian Mts, 70 km N of Kokanda, Chadak river valley | rocks | 41.00528 | 70.73917 | 25.05.1958 |  |  |  | I. A. Gubanov | MW |  |  |  |  | https://plant.depo.msu.ru/public/scan.jpg?pcode=MW0878021 |
| *Campanula alberti* | Kyrgyzstan | S slope od Chatkal range, Kassansai river valley, Terek-sai |  | 41.46806 | 71.16750 | 15.06.1976 |  |  |  | M. G. Pimenov | MW |  |  |  |  | https://plant.depo.msu.ru/public/scan.jpg?pcode=MW0878014 |
| *Campanula alberti* | Kyrgyzstan | Chatkal region, Chatkal range, Arab settl., Nayza Mt. |  | 41.57278 | 70.39389 | 29.07.1986 |  |  |  | M. G. Pimenov | MW |  |  |  |  | https://plant.depo.msu.ru/public/scan.jpg?pcode=MW0878015 |
| *Campanula alberti* | Kyrgyzstan | W Tian-Shan, N part of Chatkal range, near Jany-Bazar settl. | rocks in the supalpinian belt | 41.64269 | 70.90639 | 02.08.1962 | 2500 |  |  | V. Pavlov | MW |  |  |  |  | https://plant.depo.msu.ru/public/scan.jpg?pcode=MW0878005 |
| *Campanula alberti* | Kyrgyzstan | Chatkal river valley, upper to Sandalash river |  | 41.68444 | 70.89167 | 16.06.1976 |  |  |  | M. G. Pimenov | MW |  |  |  |  | https://plant.depo.msu.ru/public/scan.jpg?pcode=MW0878016 |
| *Campanula alberti* | Kyrgyzstan | Jalalabad Region, Chatkal District, Pskem Range (S slope), slopes on the W side of the Kara-Korum River, Kyzyl-Jar settl., ca. 14 km N of Kurulush village |  | 41.78 | 70.7175 | 08.08.2009 |  |  |  | P. Uotila | MW |  |  |  |  | https://plant.depo.msu.ru/public/scan.jpg?pcode=MW0878004 |
| *Campanula alberti* | Kyrgyzstan | Chatkal region, Pskemsky range, Santalash river bassin, Achiktash river valley |  | 41.79250 | 70.92472 | 06.08.1986 |  |  |  | M. G. Pimenov | MW |  |  |  |  | https://plant.depo.msu.ru/public/scan.jpg?pcode=MW0878013 |
| *Campanula alberti* | Kyrgyzstan | Susamyrsky range, lower part of Chichkan river, N of Toktoful settl. |  | 41.93333 | 72.86667 | 07.06.1996 | 1100 |  |  | M. G. Pimenov | MW |  |  |  |  | https://plant.depo.msu.ru/public/scan.jpg?pcode=MW0878029 |
| *Campanula alberti* | Kyrgyzstan | Jalalabad region, Naryn river basin, lower part of Chichkan river, Emkent | near the rocks | 41.95139 | 72.87778 | 21.06.2000 | 1200 |  |  | M. G. Pimenov | MW |  |  |  |  | https://plant.depo.msu.ru/public/scan.jpg?pcode=MW0878012 |
| *Campanula alberti* | Kazakhstan | Middle Asia, W. Tian-Shan, W. edge of the Talasski Range, Aksu-Jabagly Reserve, Baldybrek river valley, Ulken-darbaza | rocks | 42.27469 | 70.35308 | 25.07.1962 |  | N |  | N. Karmysheva | MW |  |  |  |  | https://www.gbif.org/occurrence/1802581573 |
| *Campanula alberti* | Kyrgyzstan | Talas Alatau, 31 km S of Kyzyl-Adyr, Kara-Buura valley |  | 42.32056 | 71.59194 | 23.07.2016 | 1720 |  |  | А. Seregin | MW |  |  |  |  | https://plant.depo.msu.ru/public/scan.jpg?pcode=MW0878003 |
| *Campanula alberti* | Kazakhstan | Talas Ala-Too Range, Aksu-Djabagly Reserve, Kshi-Kaindy River valley | rocks | 42.40167 | 70.58250 | 31.07.1948 | 3000 |  |  | I. Kultiasov | MW |  |  |  |  | https://plant.depo.msu.ru/public/scan.jpg?pcode=MW0878011 |
| *Campanula alberti* | Kazakhstan | Talas Alatau, Aksu-Djabagly Reserve, Sarkrama river valley | rocks | 42.40889 | 70.72944 | 25.07.1971 |  |  |  | E. Barabanov | MW |  |  |  |  | https://plant.depo.msu.ru/public/scan.jpg?pcode=MW0878023 |
| *Campanula alberti* | Kazakhstan | NW part of Kirgiz range, near Djambul (Taraz), meadle part of Sugaty river | rock crevices | 42.75000 | 71.82417 | 08.06.1963 |  |  |  | V. Goloskokov | MW |  |  |  |  | https://plant.depo.msu.ru/public/scan.jpg?pcode=MW0878030 |
| *Campanula alberti* | Kazakhstan | N part of Kirzgiz Alatau, Makbal range |  | 42.75306 | 72.12806 | 31.07.1930 | 2540 |  |  | G. I. Igolkin | MW |  |  |  |  | https://plant.depo.msu.ru/public/scan.jpg?pcode=MW0878032 |
| *Campanula alberti* | Kazakhstan | N part of Kirzgiz Alatau, Makbal range, Agovle-sai valley |  | 42.76944 | 72.10944 | 10.08.1930 | 2150 |  |  | G. I. Igolkin | MW |  |  |  |  | https://plant.depo.msu.ru/public/scan.jpg?pcode=MW0878034 |
| *Campanula alberti* | Kazakhstan | S Kazakhstan, Ugamsky range, Bostandyk settl, near the snow cover of Aksar-sai Mt. | rock |  |  | 28.07.1949 |  |  |  | V. Pavlov | MW |  |  |  |  | https://plant.depo.msu.ru/public/scan.jpg?pcode=MW0878007 |
| *Campanula alberti* | Uzbekistan | Arashansky region, Arashan river valley |  | 41.28889 | 70.56806 | 14.08.1924 |  |  |  | Sovetkina | TASH |  |  |  |  |  |
| *Campanula alberti* | Uzbekistan | Arashansky region, Ahangaran (Angren) and Uchsai rivers joining |  | 41.32639 | 70.62667 | 04.07.1954 |  |  |  | Nabiev, Li | TASH |  |  |  |  |  |
| *Campanula alberti* | Uzbekistan | Arashansky region, Kyzyltarsai (Kzylchasai) river valley |  | 41.36667 | 70.80000 | 15.08.1937 |  |  |  | Zokirov | TASH |  |  |  |  |  |
| *Campanula alberti* | Uzbekistan | W Chatkal region, Nurekata river valley |  | 41.41667 | 69.95000 | 17.07.1936 |  |  |  | Korotkova, Titov | TASH |  |  |  |  |  |
| *Campanula alberti* | Uzbekistan | W Chatkal region, Akbulak river valley |  | 41.51361 | 70.15222 | 04.07.1963 |  |  |  | Puchkova | TASH |  |  |  |  |  |
| *Campanula alberti* | Uzbekistan | W Chatkal region, Malyi Chimgan Mt. |  | 41.55278 | 70.03778 | 30.05.1929 |  |  |  | Gomolitskyi | TASH |  |  |  |  |  |
| *Campanula alberti* | Uzbekistan | Koksuvsky range, Paltau-sai, Burchmulla |  | 41.55333 | 70.13917 | 09.07.1921 |  |  |  | Drobov | TASH |  |  |  |  |  |
| *Campanula alberti* | Uzbekistan | Ugam range, Khumsan, Naudale Mts. |  | 41.68361 | 69.92278 | 15.07.1920 |  |  |  | Popov, Li | TASH |  |  |  |  |  |
| *Campanula alberti* | Uzbekistan | Ugam range, Bogustan settl. |  | 41.70056 | 70.11444 | 12.07.1922 |  |  |  | Simonova, Popov | TASH |  |  |  |  |  |
| *Campanula alberti* | Uzbekistan | Pskemsky range, Pskem River valley |  | 41.83333 | 70.30000 | 15.08.1946 |  |  |  | Piataeva | TASH |  |  |  |  |  |
| *Campanula alberti* | Uzbekistan | Maydantalsky range, Maydantal river valley |  | 41.98333 | 70.61667 | 16.07.1936 |  |  |  | Korotkova, Titov | TASH |  |  |  |  |  |
| *Campanula alberti* | Uzbekistan | Maydantalsky range, Oygaing river valley |  | 42.11667 | 70.88333 | 21.08.1956 |  |  |  | Tsukervanik | TASH |  |  |  |  |  |
| *Campanula alberti* | Uzbekistan | Maydantalsky range, Oygaing river basin, Tundyskai river valley |  | 42.11667 | 70.76667 | 04.06.1963 |  |  |  | Puchkova | TASH |  |  |  |  |  |
| *Campanula altaica* | Kazakhstan | Saur, Kenderlik river, left bank, way to plato | grassy meadow |  |  | 05.07.2015 |  |  |  | A. Ebiel, A. Kuprianow | KRA | 500702 |  | + | CaltC62 |  |
| *Campanula altaica* | Russia | Republic Altai, Kash-Agachsky region, near Chegan-Uzup settl., Kyzylchik river valley (left tributary of the Chegan-Uzup river) | alpine steppe |  |  | 09.08.2006 |  |  |  | A. Ebiel | KRA | 500701 |  | + | CaltC84 |  |
| *Campanula cervicaria* | Poland | Osiny near Mirzec, department 18/21, forest inspectorate Marcule | mixed forest |  |  | 31.08.2018 |  |  |  | M. Nobis | KRA | 486067 |  | + | Ccer76 |  |
| *Campanula dasyantha* | Mongolia | Aimak Central, somon Batsumber, Chentei Mts, the main ridge in the vicinity of Asaraltu | high mountain grassland |  |  | 18.07.1977 | 2100 |  |  | A. Pacyna | KRA | 100388 |  | + | Cdas75 |  |
| *Campanula glomerata* | Kyrgyzstan | Central Tian-Shan, ca. 6.5 km NNW of At-Bashy, ca. 36 km SW of Naryn | saline roadside (left side towards At-Bashy) | 41.18185 | 75.72175 | 06.07.2018 | 2041 |  |  | M. N., E. K., A. W., A. N. | KRA | 500738 |  | + | Cglo98 |  |
| *Campanula glomerata* | Kyrgyzstan |  |  | 42.2516111 | 73.04475 | 2017 |  |  |  | M. N., E. K., A. W., A. N. | KRA (gel) |  |  | + | Cglo196 |  |
| *Campanula incanescens* | Tajikistan | Gorno-Badakhshan Autonomous Region, Western Pamir, Panj river valley, ca. 64 km SE of Kalaikhum, ca. 67 km NNW of Khorog |  | 38.055171 | 71.311203 | 2019.07.25 | 1681 |  |  | E. K., A. W. | KRA (gel) |  |  | + | Cinc1370/4 |  |
| *Campanula incanescens* | Tajikistan | Gorno-Badakhshan Autonomous Region, Darvaz Range, Panj river valley, ca. 105 km NE of Vose, ca. 161 km E of Dushanbe |  | 38.344088 | 70.609841 | 2019.07.24 | 1170 |  |  | E. K., A. W. | KRA (gel) |  |  | + | Cinc1363/4 |  |
| *Campanula persicifolia* | Poland | Dąbie natural reserve |  |  |  | 15.06.2017 |  |  |  | M. N., E. K. | KRA (gel) |  |  | + | Cper200 |  |
| *Campanula rapunculoides* | Poland | Kalina Lisiniec |  |  |  | 15.06.2017 |  |  |  | M. N., E. K. | KRA (gel) |  |  | + | Crap186 |  |
| *Campanula rapunculoides* | Poland | Pińczów |  |  |  | 15.06.2017 |  |  |  | M. N., E. K. | KRA (gel) |  |  | + | Crap197 |  |
| *Campanula sibirica* | Poland | Dąbie natural reserve |  |  |  | 15.06.2017 |  |  |  | M. N., E. K. | KRA (gel) |  |  | + | Csib198 |  |
| *Campanula sibirica* | Poland | Dąbie natural reserve |  |  |  | 15.06.2017 |  |  |  | M. N., E. K. | KRA (gel) |  |  | + | Csib199 |  |
| *Campanula trachelium* | Poland | Kwiatówka |  |  |  |  |  |  |  | M. N., E. K. | KRA (gel) |  |  | + | Ctra102 |  |
| *Campanula turczaninovii* | Mongolia | Mungun-Moritu, Chentei, Dund-Baidauagiju-Gol, NNE part, N of mountain slope | forest glade |  |  | 23.07.1978 | 1600 |  |  | F. Święs | KRA | 101199 |  | + | Ctur103 |  |
| *Campanula turczaninovii* | Mongolia | Ajmak Bajanchongor, somon Galuut, Changai Mts, Sant valley (side from Cagan-Turutuin-gol) | scrub |  |  | 30.06.1974 | 2530 |  |  | A. Pacyna | KRA | 101273 |  | + | Ctur104 |  |
| *Campanula  lehmanniana (C. eugeniae)* | Kyrgyzstan | Central Tian-Shan ca. 25 km NNW of Toktogul, ca. 97 km W of Suusamyr | rocks | 42.08296 | 72.80968 | 30.06.2015 | 1595 |  |  | M. N., A. N. | KRA | 500726 |  | + | Ceug253 |  |
| *Campanula  lehmanniana (C. eugeniae)* | Tajikistan | 7'lake valley, Marguzor |  | 39.16500 | 67.83972 | 2015 |  |  |  | M. N., A. N. | KRA (gel) |  |  | + | Cleh212 |  |
| *Campanula  lehmanniana (C. eugeniae)* | Tajikistan | Anzob |  | 39.17361 | 68.78833 | 31.05.2015 |  |  |  | M. N., A. N. | KRA (gel) |  |  | + | Cleh203 |  |
| *Campanula  lehmanniana (C. eugeniae)* | Kyrgyzstan | Kyrgyzstan, ca. 23 km N of Toktogul Lake |  | 42.05945 | 72.81888 | 2017 |  |  |  | M. N. | KRA (gel) |  |  | + | Ceug159 |  |
| *Sergia regelii* | Tajikistan | Zeravshan Mts (Pamir Alai), left slope of Archamaidan river valley, ca. 6 km S of Zimtut settl. | rock grassland | 39.146583 | 68.035944 | 17.06.2010 | 2300 | E | 85 | M. N., M. Kozak | KRA | 463618 | + |  |  |  |
| *Sergia regelii* | Tajikistan | Sughd Region, Fann Range, ca. 49 km SW of Aini, ca. 49 km SE of Panjakent | rocks | 39.185828 | 68.02395 | 20.06.2019 | 2125 | E | 85 | M. N., J. Zalewska-Gałosz, E. K., A. W. | KRA | 522679 | + |  |  |  |
| *Sergia regelii* | Tajikistan | Pamir Alai Mts, Zeravshan Mts, Lake nr 1, 7 Lakes Valley | rocks | 39.219722 | 67.800139 | 10.06.2010 | 1440 |  |  | M. N. | KRA | 522693 | + |  |  |  |
| *Sergia regelii* | Tajikistan | Zeravshan Mts (Pamir Alai), Kshtut river valley, Archamidon river gorge (left slope), among Gazza and Zimtut settl. | rock grassland, rock crevices | 39.267222 | 68.048333 | 14.06.2010 | 1560 | NEE | 85 | M. N. | KRA | 452874 |  |  |  |  |
| *Sergia regelii* | Tajikistan | Sughd Region, Kshtut River valley, ca. 3 km S of Gaza | scree in the river valley | 39.276667 | 68.049722 | 27.05.2015 | 1590 |  |  | M. N. | KRA | 500718 | + | + | Sereg1 |  |
| *Sergia regelii* | Tajikistan | Zeravshan Mts (Pamir Alai), Uriech river gorge, ca. 3 km S of Jakahona settl. (ca. 3 km N of Artuch basecamp) | rock crevices | 39.283333 | 68.116667 | 26.06.2008 | 2200 | W | 80 | M. N. | KRA | 459197 | + |  |  |  |
| *Sergia regelii* | Turkmenistan | Ketman-Chanty pass, Tapa plateau | rocks | 37.647139 | 66.517694 | 25.08.1958 |  |  |  | Vasilchenko et al. | LE | 364 | + |  |  |  |
| *Sergia regelii* | Turkmenistan | Kugitang (Koitendag) range, Hojaypil (Khodzhapil), valley with spring |  | 37.868333 | 66.60675 | 16.06.1966 |  |  |  | A. I. Meshanikov et al. | LE |  | + |  |  |  |
| *Sergia regelii* | Turkmenistan | Kugitang (Koitendag) range, Kugitang (Koyten) settl. | rocky crevices | 37.885289 | 66.496031 | 23.08.1928 | 2700 |  |  | E. Bobrov | LE | 1471 | + |  |  |  |
| *Sergia regelii* | Turkmenistan | Kugitang (Koitendag) range, Kizyl-alma |  | 37.898056 | 66.60675 | 28.06.1927 |  |  |  | Popov, Vvedensky | LE |  | + |  |  |  |
| *Sergia regelii* | Turkmenistan | Kugitang (Koitendag) Range, near Hodja-pil ata settl, Emerdara canyon |  | 37.933056 | 66.669 | 04.06.1982 |  |  |  | R. Kamelin et al. | LE | 759 | + |  |  |  |
| *Sergia regelii* | Uzbekistan | Kugitang (Koitendag) range, Tangiduval Mts | rock crevices | 37.989469 | 66.785072 | 13.06.1971 |  |  |  | V. Bochancev | LE | 131 | + |  |  |  |
| *Sergia regelii* | Uzbekistan | Boysun, Avlad settl | rocks | 38.278278 | 67.186306 | 03.08.1940 |  |  |  | Kudriashev | LE | 285 | + |  |  |  |
| *Sergia regelii* | Uzbekistan | Baisun Mts, left bank of the Machai-darya river, between Ukarimachai and Machaimien settl. |  | 38.35153 | 67.09407 | 08.06.1982 |  |  |  | R. Kamelin et al. | LE | 929 |  | + | Sereg5 |  |
| *Sergia regelii* | Uzbekistan | S Hissar range, Sangardak Nilu |  | 38.604444 | 67.530556 | 22.05.1913 |  |  |  | A.I. Mihelson | LE | 3653 | + |  |  |  |
| *Sergia regelii* | Uzbekistan | W Pamir Alai Mts, upper part of Jakkabag-darya river, left bank, W of Tash-Kurgan settl | calcaeous rocks | 38.761525 | 67.210619 | 27.06.1936 |  |  |  | V. Bochancev, A. Butkov | LE | 494 | + |  |  |  |
| *Sergia regelii* | Uzbekistan | W Hissar Mts, Aksu river valley, between Gisarak and Kzyl-Emchek settl. | rocks | 38.861389 | 67.798844 | 11.06.1982 |  |  |  | R. Kamelin et al. | LE | 1124 | + |  |  |  |
| *Sergia regelii* | Uzbekistan | W Hissar range, Osmontala Mt. | rock crevices | 38.9026 | 67.423936 | 10.07.1961 | 2800 |  |  | Miriakin | LE |  | + |  |  |  |
| *Sergia regelii* | Uzbekistan | W Hissar Mts, Kashkadarya-Aksu river basins, upper to Mirоki settl., Chakky valley |  | 39.056067 | 67.30275 | 11.06.1982 |  |  |  | R. Kamelin et al. | LE | 1021 | + |  |  |  |
| *Sergia regelii* | Uzbekistan | Samarkand Region, Jakka-Chana (up to Kshtut) |  | 39.074028 | 67.343 | 24.06.1899 |  |  |  | V. Lipsky | LE | 3499 | + |  |  |  |
| *Sergia regelii* | Uzbekistan | W Hissar Mts, Kashkadarya-Aksu river basins, upper part of Gelandarya river, righ bank | dry hills | 39.085917 | 67.425611 | 13.06.1982 |  | S and E |  | R. Kamelin et al. | LE | 1242 | + |  |  |  |
| *Sergia regelii* | Tajikistan | Zeravshan Mts, Marguzor and Hazor-Chashma valleys |  | 39.1045 | 67.862028 | 28.07.1931 |  |  |  | S. A. Nikitin | LE | 665 | + |  |  |  |
| *Sergia regelii* | Tajikistan | W slope of Chazr Sultan, between Mogiyon and Darai-kalen rivers, Maslahat-tepe |  | 39.197139 | 67.740139 | 10.06.1913 |  |  |  | A. I. Mihelson | LE | 3655 | + |  |  |  |
| *Sergia regelii* | Tajikistan | W Zeravshan Mts, Nejigon (Mijgon) lake (Marguzarsky lakes) | rocky hill | 39.228914 | 67.800297 | 16.07.1932 |  |  |  | P. Ovchinnikov, A. Slobodov | LE | 1100 |  |  |  |  |
| *Sergia regelii* | Tajikistan | Fann Mts, near Kulikalon lake, rocks up to lake | rocks | 39.249017 | 68.174542 | 08.1965 |  |  |  | R. Kamelin | LE |  | + |  |  |  |
| *Sergia regelii* | Tajikistan | N hill of Zeravshan Mts, 1.5 km W of Ghorif settl. |  | 39.273194 | 69.986583 | 28.06.1961 | 2300 |  |  | E. I. Filatov, V. N. Zavedeev | LE | 92 | + |  |  |  |
| *Sergia regelii* | Uzbekistan | Samarkand Mts, lower to Amhankutan settl. |  | 39.280953 | 66.961806 | 17.06.1982 |  |  |  | R. Kamelin et al. | LE | 1565 | + |  |  |  |
| *Sergia regelii* | Tajikistan | Zeravshan Mts, Zimtut |  | 39.29 | 68.05 | 20.07.1913 |  |  |  | L. Borimuller | LE | 195 |  |  |  |  |
| *Sergia regelii* | Tajikistan | Fann Mts, from Kulikalon lake to Kshtut river valley, 5 km up to Pajrut settl. | rocks | 39.306052 | 68.114725 | 08.1965 |  |  |  | R. Kamelin | LE |  | + |  |  |  |
| *Sergia regelii* | Tajikistan | Samarkand province, near Farob |  | 39.308861 | 67.384694 | 17.06.1912 |  |  |  | I. A. Preobrazhensky | LE |  | + |  |  |  |
| *Sergia regelii* | Tajikistan | W Zeravshan Mts, W of Pagna settl. | rocks | 39.325638 | 68.017442 | 20.06.1932 | 2200 | N |  | P. Ovchinnikov, A. Slobodov | LE | 763 | + |  |  |  |
| *Sergia regelii* | Tajikistan | Zeravshan Mts, Artuch-Darya river valley, Artuch |  | 39.335806 | 68.112106 | 17.06.1913 |  |  |  | A. I. Mihelson | LE | 2572 | + |  |  |  |
| *Sergia regelii* | Uzbekistan | Zeravshan Mts, Urgut settl., Fara-chinar settl. | rock crevices | 39.337333 | 67.246028 | 06.06.1936 |  |  |  | A. Gnezdillo | LE | 157 | + |  |  |  |
| *Sergia regelii* | Tajikistan | N Zeravshan Mts, left bank of Kshtut river, Negnot settl. | rock crevices | 39.353814 | 68.024814 | 05.08.1963 | 2400 |  |  | S. Upusov et al. | LE | 6745 | + |  |  |  |
| *Sergia regelii* | Tajikistan | Zeravshan Mts, right bank of Kshtut river, between Kuliali and Shishkat settl. | rock crevices | 39.369083 | 68.067972 | 27.06.1961 |  | E |  | T. Strizhova, R. Kamelin | LE | 737 | + |  |  |  |
| *Sergia regelii* | Tajikistan | N Zeravshan Mts, left bank of Kshtut river, between Kuliali and Shishkat settl. | rock crevices | 39.378769 | 68.041313 | 27.06.1961 |  |  |  | T. Strizhova, R. Kamelin | LE | 737 |  |  |  |  |
| *Sergia regelii* | Tajikistan | Zeravshan Mts, Kshtut river, Kshtut settl. |  | 39.46 | 68.12 | 14.06.1932 |  |  |  | P. Ovchinnikov, A. Slobodov | LE | 576 |  |  |  |  |
| *Sergia regelii* | Tajikistan | Turkestan range, Aftobruia, Ui-bodom settl |  | 39.74 | 69.28 | 21.07.1926 |  |  |  | P. S. Massagetov | LE | 21 | + |  |  |  |
| *Sergia regelii* | Uzbekistan | Malguzar, Pshigar valley |  | 39.960027 | 68.115859 | 16.06.1914 |  |  |  | A. I. Mihelson | LE |  |  |  |  |  |
| *Sergia regelii* | Uzbekistan | Buhara, Jakkabag |  | 39.966667 | 68.666667 | 18.06.1896 |  |  |  | V. I. Lipsky | LE |  |  |  |  |  |
| *Sergia regelii* |  | Shut, Katha-darvi |  |  |  | 1893 |  |  |  | V. L. Komarov | LE |  |  |  |  |  |
| *Sergia regelii* | Turkmenistan/Uzbekistan | Kugitang range, Tash-Dara valley | rocks |  |  | 11.05.1915 |  |  |  | M. T. Popova | LE | 11690 |  |  |  |  |
| *Sergia regelii* | Uzbekistan/Tajikistan | Zeravshan Mts, Schink |  |  |  | 30.05.1892 |  |  |  | V. L. Komarov | LE |  |  |  |  |  |
| *Sergia regelii* | Uzbekistan | Buhara, Hissar Mts, Chatg valley |  |  |  | 12.06.1897 |  |  |  | V. Lipsky | LE | 3498 |  |  |  |  |
| *Sergia regelii* | Uzbekistan/Tajikistan | S hill of Hissar Mts, upper part of Hoveet river |  |  |  | 24.09.1928 | 3200 |  |  | N. Gontscharow | LE | 2083 |  |  |  |  |
| *Sergia regelii* | Tajikistan | Zeravshan Mts, Woru river |  | 39.243906 | 67.974539 | 06.07.1882 | 2785 |  |  | A. Regel | MNHN |  | + |  |  | https://www.gbif.org/ru/occurrence/438643068 |
| *Sergia regelii* | Uzbekistan | Hissar range, Minkuchar | rocks | 39.127872 | 67.263178 | 18.08.1935 | 3100 |  |  | P. Gordienko | MW |  | + |  |  | https://www.gbif.org/ru/occurrence/1799052579 |
| *Sergia regelii* | Tajikistan | Voru (Kshtut-Darya) river valley | rocks | 39.243447 | 67.960197 | 01.08.1932 | 1650 |  |  | I. A. Gcukiny | MW |  | + |  |  | https://www.gbif.org/ru/occurrence/1799052615 |
| *Sergia regelii* | Tajikistan | W slope of Pmiro Alai Mts, Shing river valley (left tributary of Mogiendarya river) | rocks | 39.285806 | 67.864833 | 1963 | 2063 |  |  | I. S. Schukin | MW |  | + |  |  | https://www.gbif.org/ru/occurrence/1799052608 |
| *Sergia regelii* | Uzbekistan | Zeravshan range, Kyrk-Tau, Akbai-Djuma | rocks | 39.317972 | 67.241825 | 29.06.1933 | 2500 |  |  | P. Gordienko, A. Chilina | MW |  | + |  |  | https://www.gbif.org/ru/occurrence/1799052637 |
| *Sergia regelii* | Uzbekistan | N slope of Zeravshan range, up to Urgut settl. |  | 39.365203 | 67.242414 | 30.05.1978 | 1545 |  |  | M. G. Pimenov et al. | MW |  | + |  |  | https://www.gbif.org/ru/occurrence/1799052649 |
| *Sergia regelii* | Uzbekistan | Djizaksk Region, S slope of Malguzarsky range, beside Bahmal settl, Shurbaly pass |  | 39.828667 | 67.959194 | 04.07.1991 | 2425 |  |  | M. G. Pimenov et al. | MW |  | + |  |  | https://www.gbif.org/ru/occurrence/1799052634 |
| *Sergia regelii* | Uzbekistan | Kashka-Darya Region, upper part of Tanhaz-Darya river, near Shurkhasan settl., Beshnau Mts, Kainarsai |  | 39.909722 | 67.251389 | 29.06.1988 | 1409 |  |  | M. G. Pimenov et al. | MW |  | + |  |  | https://www.gbif.org/ru/occurrence/1799052602 |
| *Sergia regelii* | Uzbekistan | Kuhitang region, Kyzylalma |  | 37.8429 | 66.7130 | 28.06.1927 |  |  |  | Popov | TASH |  |  |  |  |  |
| *Sergia regelii* | Uzbekistan | Kashkadarya region, Kyzyltam, way to Igrisu |  | 38.6477 | 67.1021 | 26.07.1942 |  |  |  | Korotkova | TASH |  |  |  |  |  |
| *Sergia regelii* | Tajikistan | Zeravshan Mts, 7th Lakes valley, NE bank of 7th Lake |  | 39.1150000 | 67.8533333 |  | 2394 | NE | 90 | A. N. et al. - field observation |  |  | + |  |  |  |
| *Sergia regelii* | Tajikistan | Zeravshan Mts, Archamaydon river valley, ca. 13 km SW of Voru |  | 39.1300000 | 68.0697222 |  | 2337 | S | 45 | A. N. et al. - field observation |  |  | + |  |  |  |
| *Sergia regelii* | Tajikistan | Zeravshan Mts, Khshirt river valley, ca. 3.7 km SW of Marzich |  | 39.153611 | 68.7700000 |  | 2798 | N | 80 | A. N. et al. - field observation |  |  | + |  |  |  |
| *Sergia regelii* | Tajikistan | Zeravshan Mts, Archamaydon river valley, ca. 9 km SW of Voru |  | 39.153888 | 68.0288888 |  | 2118 | SW | 45 | A. N. et al. - field observation |  |  | + |  |  |  |
| *Sergia regelii* | Tajikistan | Zeravshan Mts, 7th Lakes valley, SW bank of 5th Lake (Khurdak) |  | 39.165277 | 67.8400000 |  | 1926 | W | 75 | A. N. et al. - field observation |  |  | + |  |  |  |
| *Sergia regelii* | Tajikistan | Zeravshan Mts, Archamaydon river valley, ca. 5.5 km SW of Voru |  | 39.189166 | 68.0261111 |  | 1866 | W | 85 | A. N. et al. - field observation |  |  |  |  |  |  |
| *Sergia regelii* | Tajikistan | Zeravshan Mts, 7th Lakes valley, NW bank of 4th Lake (Nofin) |  | 39.189444 | 67.8308333 |  | 1802 | W | 85 | A. N. et al. - field observation |  |  | + |  |  |  |
| *Sergia regelii* | Tajikistan | Zeravshan Mts, 7th Lakes valley, NW bank of 4th Lake (Nofin) |  | 39.189722 | 67.8305555 |  | 1805 | NW | 90 | A. N. et al. - field observation |  |  |  |  |  |  |
| *Sergia regelii* | Tajikistan | Zeravshan Mts, 7th Lakes valley, ca. 1 km E of 4th Lake (Nofin) |  | 39.191111 | 67.8080510 |  | 2478 | W | 85 | A. N. et al. - field observation |  |  |  |  |  |  |
| *Sergia regelii* | Tajikistan | Zeravshan Mts, 7th Lakes valley, NW bank of 4th Lake (Nofin) |  | 39.191388 | 67.8255555 |  | 1792 | NW | 80 | A. N. et al. - field observation |  |  |  |  |  |  |
| *Sergia regelii* | Tajikistan | Zeravshan Mts, 7th Lakes valley, E bank of 3th Lake (Hushyor) |  | 39.205000 | 67.8080555 |  | 1800 | NE | 90 | A. N. et al. - field observation |  |  | + |  |  |  |
| *Sergia regelii* | Tajikistan | Zeravshan Mts, 7th Lakes valley, E bank of 3th Lake (Hushyor) |  | 39.205277 | 67.8077777 |  | 1799 | NW | 10 | A. N. et al. - field observation |  |  |  |  |  |  |
| *Sergia regelii* | Tajikistan | Zeravshan Mts, 7th Lakes valley, E bank of 3th Lake (Hushyor) |  | 39.205555 | 67.8080555 |  | 1756 | NE | 15 | A. N. et al. - field observation |  |  |  |  |  |  |
| *Sergia regelii* | Tajikistan | Zeravshan Mts, ca. 19 km W of Voru |  | 39.221388 | 68.2058333 |  | 3520 | W | 80 | A. N. et al. - field observation |  |  |  |  |  |  |
| *Sergia regelii* | Tajikistan | Zeravshan Mts, ca. 19 km W of Voru |  | 39.228055 | 68.2058333 |  | 3184 | W | 80 | A. N. et al. - field observation |  |  |  |  |  |  |
| *Sergia regelii* | Tajikistan | Zeravshan Mts, ca. 5 km W of Voru |  | 39.230833 | 68.0419444 |  | 2164 | SWW | 85 | A. N. et al. - field observation |  |  | + |  |  |  |
| *Sergia regelii* | Tajikistan | Zeravshan Mts, ca. 2.3 km W of Voru |  | 39.231388 | 68.0127777 |  | 1902 | SWW | 70 | A. N. et al. - field observation |  |  | + |  |  |  |
| *Sergia regelii* | Tajikistan | Zeravshan Mts, Moghiyon |  | 39.237500 | 67.6416666 |  | 1615 | NE | 80 | A. N. et al. - field observation |  |  | + |  |  |  |
| *Sergia regelii* | Tajikistan | Zeravshan Mts, Kshtut river valley, ca. 2 km W of Negnot |  | 39.323333 | 68.0316666 |  | 1464 | NW | 90 | A. N. et al. - field observation |  |  | + |  |  |  |
| *Sergia regelii* | Tajikistan | Zeravshan Mts, Kshtut river valley, ca. 4 km SW of Negnot |  | 39.365000 | 68.0386111 |  | 1359 | NE | 85 | A. N. et al. - field observation |  |  | + |  |  |  |
| *Sergia regelii* | Tajikistan | Zeravshan Mts, ca. 9 km S of Hussar |  | 39.378611 | 67.8475000 |  | 1861 | W | 90 | A. N. et al. - field observation |  |  | + |  |  |  |
| *Sergia regelii* | Tajikistan | Zeravshan Mts, ca. 9 km S of Hussar |  | 39.379444 | 67.8477777 |  | 1778 | N | 85 | A. N. et al. - field observation |  |  |  |  |  |  |
| *Sergia regelii* | Uzbekistan | Sangardak waterfall |  | 38.535278 | 67.563333 | 11.06.2013 | 1307 |  |  | A. Yakovlev |  |  | + |  |  | https://www.plantarium.ru/page/image/id/195545.html |
| *Sergia regelii* | Uzbekistan | Kaszkadarya obl, Kyzyl-su river, kalasai valley |  | 38.749722 | 67.277222 | 01.06.2013 | 2158 |  |  | N. Beshko |  |  | + |  |  | https://www.plantarium.ru/page/image/id/204737.html |
| *Sergia regelii* | Uzbekistan | Besh-nau Mts |  | 38.790833 | 67.115 | 20.07.2013 | 1817 |  |  | T. Tillaev |  |  | + |  |  | https://www.plantarium.ru/page/image/id/226297.html |
| *Sergia regelii* | Tajikistan | Zeravshan Mts, ca. 12 km SW of Voru |  | 39.141389 | 68.08 |  | 2776 | E | 85 | A. N. et al. - field observation |  |  | + |  |  |  |
| *Sergia regelii* | Tajikistan | Zeravshan Mts, 7th Lakes valley, NW bank of 4th Lake (Nofin) |  | 39.19222 | 67.8247222 |  | 1793 | W | 90 | A. N. et al. - field observation |  |  |  |  |  |  |
| *Sergia regelii* | Tajikistan | Zeravshan Mts, ca. 19 km N of Voru |  | 39.239028 | 68.201669 |  | 2996 | W | 80 | A. N. et al. - field observation |  |  | + |  |  |  |
| *Sergia regelii* | Tajikistan | Zeravshan Mts, ca. 18 km W of Voru |  | 39.253611 | 68.409444 |  | 2267 | SW | 90 | A. N. et al. - field observation |  |  | + |  |  |  |
| *Sergia regelii* | Tajikistan | Zeravshan Mts, ca. 10 km NW of Aini |  | 39.308056 | 68.488056 |  | 2152 | SW | 80 | A. N. et al. - field observation |  |  | + |  |  |  |
| *Sergia regelii* | Tajikistan | Zeravshan Mts, ca. 13 km NW of Artuch |  | 39.352778 | 68.242778 |  | 2613 | SE | 85 | A. N. et al. - field observation |  |  | + |  |  |  |
| *Sergia sewerzowii* | Kazakhstan | Aksu-Djabagly reserve, Aksu river |  | 42.303056 | 70.57 | 09.07.1974 | 1716 |  |  | R. Kamelin | LE | 1646 | + | + | Sesew4 |  |
| *Sergia sewerzowii* | Kazakhstan | Talass Alatau, Aksu river valley, Mansura | rocks | 42.337778 | 70.488056 | 25.07.1933 | 1738 |  |  | I. A. Linchaevsky | LE | 493 | + |  |  |  |
| *Sergia sewerzowii* | Kazakhstan | Aksu-Djabagly reserve, Jetysai valley |  | 42.373056 | 70.586111 | 24.07.1958 | 2392 |  |  | L. P. Markova, L. I. Medvedeva | LE |  | + |  |  |  |
| *Sergia sewerzowii* | Kazakhstan | Keltemashat, Mashat river |  | 42.405 | 70.289278 | 15.06.1924 | 1200 |  |  | E. A. Mokiv | LE | 989 | + |  |  |  |
| *Sergia sewerzowii* | Kazakhstan | W Tian Shan, Mashatsky Mts, Uzun-Mashat (Mashat) river valley | rocks | 42.429167 | 70.301389 | 17.07.1958 | 1348 |  |  | L. P. Markova, L. I. Medvedeva | LE |  | + | + | Sesew6 |  |
| *Sergia sewerzowii* | Kazakhstan | Chimkent province, middle part of Mashat river, Mashat settl |  | 42.433889 | 70.012778 | 20.06.1921 | 730 |  |  | R. I. Abolin | LE | 7673 | + |  |  |  |
| *Sergia sewerzowii* | Kazakhstan | Talass Alatau, Seslavino settl. | rocks | 42.441056 | 70.248611 | 09.06.1951 | 1395 |  |  | I. I. Roldugin | LE |  | + |  |  |  |
| *Sergia sewerzowii* | Kazakhstan | Karatau Mts, Boztorgai valley | rocky hill with bushes | 42.69325 | 70.266111 | 19.07.1950 | 980 |  |  | A. I. Pojarkova | LE | 255 | + |  |  |  |
| *Sergia sewerzowii* | Kazakhstan | Karatau Mts, Saja-su river valley |  | 42.8525 | 70.837325 | 25.06.1931 | 708 |  |  | N. V. Pavlov | LE | 351 | + |  |  |  |
| *Sergia sewerzowii* | Kazakhstan | Syrdarynsky Karatau, Bilikol lake, Berkaza gorge |  | 42.906453 | 70.619847 | 27.08.1969 | 837 |  |  | R. Kamelin | LE |  | + |  |  |  |
| *Sergia sewerzowii* | Kyrgyzstan | Talass Alatau, Taldy-bulak river | rocky hill | 42.04056 | 71.59722 | 07.07.1958 | 2264 | W |  | V. N. Golubev | MW | 880924 | + |  |  | https://www.gbif.org/ru/occurrence/1799052622 |
| *Sergia sewerzowii* | Kazakhstan | S Kazakhstan, Aksu-Djabagly reserve, Aksu river valley | rocky crevices | 42.321667 | 70.554722 | 14.07.1961 | 1737 |  |  | E. Barabanov, E. Kurchenko | MW | 880917 | + |  |  | https://www.gbif.org/ru/occurrence/1799052628 |
| *Sergia sewerzowii* | Kazakhstan | S Kazakhstan, near Tulkubas, Duona-tau Mts, Daubaba valley |  | 42.421389 | 70.2625 | 08.08.1982 | 1112 |  |  | M. G. Pimenov et al. | MW | 880916 | + |  |  | https://www.gbif.org/ru/occurrence/1799052592 |
| *Sergia sewerzowii* | Kazakhstan | Syr-Darya province, Mashat Mts, oposite to Tulkubas station | rocky hill | 42.469308 | 70.286367 | 02.07.1934 | 1089 |  |  | N. V. Pavlov | MW | 880925 | + |  |  | https://www.gbif.org/ru/occurrence/1799052554 |
| *Sergia sewerzowii* | Kazakhstan | near Mashat railway station |  | 42.510833 | 69.912156 | 12.06.1924 | 527 |  |  | P. Gomoyancky | MW | 880920 | + |  |  | https://www.gbif.org/ru/occurrence/1799052632 |
| *Sergia sewerzowii* | Kazakhstan | near Mashat railway station | rocks | 42.705694 | 70.287222 | 23.07.1936 | 1113 |  |  | L. Chilikina | MW | 880929 | + |  |  | https://www.gbif.org/ru/occurrence/1799052598 |
| *Sergia sewerzowii* | Kazakhstan | Karatau Mts, Belbulak river | rocky crevices | 42.8925 | 70.611667 | 30.06.1931 | 916 |  |  | N. V. Pavlov | MW | 880918 | + |  |  | https://www.gbif.org/ru/occurrence/1799052653 |
| *Sergia sewerzowii* | Kazakhstan | Karatau Mts, Kanym-sai | rocky hill |  |  | 17.06.1934 |  | SE |  | Hibnokova | MW | 880914 |  |  |  | https://www.gbif.org/ru/occurrence/1799052580 |
| *Sergia sewerzowii* | Kazakhstan | Southern Kazakhstan, Karakus Mountains |  | 42.506667 | 70.121389 |  | 1281 |  |  | E. Davkaev |  |  | + |  |  | http://www.fotomontaro.com/flora/campanula/sergia_sewerzowii06.shtml |
| *Sergia sewerzowii* | Kazakhstan | Southern Kazakhstan, Boroldaytau Range, Kokbulak Valley |  | 42.732333 | 70.185056 |  | 1202 |  |  | E. Davkaev |  |  | + |  |  | http://www.fotomontaro.com/flora/campanula/sergia_sewerzowii02.shtml |
| *Sergia sewerzowii* | Kazakhstan | Turkistan Region (formerly South Kazakhstan Region), Tulkibas District, surroundings of Aksay village, valley of Mashat river |  | 42.41874 | 70.25635 | 10.06.2017 | 1040 |  |  | A. Ebel - field observation |  |  |  |  |  |  |

**Supplementary Table S3.** The results of the niche overlap analyses (similarity and equivalence tests p values for niche conservatism (“greater”) and divergence (“lower”) hypotheses. Statistically significant p-values are in bold.

| Niche overlap | | D index | I index |
| --- | --- | --- | --- |
|  |  | 0.0085 | 0.0505 |
| niche conservatism hypothesis | similarity (p) | 0.1678 | 0.1089 |
|  | equivalency (p) | 0.9703 | 1 |
| niche divergence hypothesis | similarity (p) | 0.8701 | 0.8871 |
|  | equivalency (p) | **0.0495** | **0.0099** |

**Supplementary Table S4.** Changes in the potential distribution area (km^2^) for *S. regelii* and *S. sewerzowii* under the future climate scenarios.

| Climate scenarios | *Sergia regelii* | | | *Sergia sewerzowii* | | |  |
| --- | --- | --- | --- | --- | --- | --- | --- |
|  | Loss | Gain | Unchanged | Loss | Gain | Unchenged | |
| RPC2.6 (2041-2060) | 8,983.1 | 59.6 | 18,243.3 | 2,899.8 | 212.7 | 11,654.1 | |
| RPC2.6 (2061-2080) | 8,745.8 | 77.5 | 18,480.6 | 908.6 | 2,830.8 | 13,645.3 | |
| RPC8.5 (2041-2060) | 11,451.8 | 73.7 | 15,774.6 | 2,428.0 | 2,474.4 | 12,125.9 | |
| RPC8.5 (2061-2080) | 17,574.8 | 53.9 | 9,651.6 | 1,499.5 | 8,127.4 | 13,054.4 | |

**Supplementary Table S5.** Primers used for amplification and sequencing reactions.

| region | primers | sequence | authors |
| --- | --- | --- | --- |
| psbD-trnT | psbD | CTC CGT ARC CAG TCA TCC ATA | (Shaw, Lickey, Schilling, & Small, 2007) ^4^ |
|  | trnT(GGU)-R | CCC TTT TAA CTC AGT GGT AG |  |
| rpl16 | rpl16F | CTATGCTTAGTGTGTGACTC | (Löhne, Borsch, &  Wiersema, 2007) ^5^ |
|  | rpl16R | TCTTCCTCTATGTTGTTTACG | (Campagna & Downie, 1998) ^6^ |
| trnS-trnfM | trnS | GAGAGAGAGGGATTCGAACC | (Demesure, Sodzi, & Petit, 1995) ^7^ |
|  | trnfM | CATAACCTTGAGGTCACGGG |  |
| petD | PIpetB1411F | GCCGTMTTTATGTTAATGC | (Löhne & Borsch, 2005) ^8^ |
|  | PIpetD738R | AATTTAGCYCTTAATACAGG |  |
| trnK-psbA | trnK-F | GGGTTGCTAACTCAATGGTAGAG | (Wicke & Quandt, 2009) ^9^ |
|  | psbA5'R | ACCATCCAATGTAAAGACGGTTT | (Shaw et al., 2005) ^10^ |

**Supplementary Table S6.** Environmental variables tried as predictors for modeling the potential distribution of *S. regelii* and *S. sewerzowii*. Variables selected for modeling are in bold.

| Code | Environmental variables | Code | Environmental variables |
| --- | --- | --- | --- |
| bio1 | Annual Mean Temperature, °C | bio18 | Precipitation of Warmest Quarter, mm |
| bio2 | Mean Diurnal Range (Mean of monthly (max temp - min temp)), °C | **bio19** | **Precipitation of Coldest Quarter, mm** |
| bio3 | Isothermality (BIO2/BIO7) x 100 | **elev** | **Elevation, m** |
| bio4 | Temperature Seasonality (standard deviation x 100), °C | sra | Average annual solar radiation, W/m2 |
| bio5 | Max Temperature of Warmest Month, °C | **srh** | **Average solar radiation in a quarter with highest solar radiation, W/m2** |
| bio6 | Min Temperature of Coldest Month, °C | **srl** | **Average solar radiation in a quarter with lowest solar radiation, W/m2** |
| **bio7** | **Temperature Annual Range (BIO5-BIO6), °C** | awc | Available soil water capacity |
| **bio8** | **Mean Temperature of Wettest Quarter, °C** | bld | Bulk density (fine earth), kg/m^3^ |
| bio9 | Mean Temperature of Driest Quarter, °C | **cec** | **Cation Exchange Capacity of soil, cmolc/kg** |
| bio10 | Mean Temperature of Warmest Quarter, °C | ocd | Soil organic carbon density, kg Carbon/m2 |
| bio11 | Mean Temperature of Coldest Quarter, °C | **ocs** | **Soil organic carbon stock, t/ha** |
| bio12 | Annual Precipitation, mm | occ | Soil organic carbon content, g/kg |
| bio13 | Precipitation of Wettest Month, mm | **ph** | **pH index measured in water solution** |
| bio14 | Precipitation of Driest Month, mm | cly | Weight percentage of the clay particles (<0.0002 mm), % |
| bio15 | Precipitation Seasonality (Coefficient of Variation) | **crf** | **Volumetric percentage of coarse fragments (>2 mm), %** |
| bio16 | Precipitation of Wettest Quarter, mm | slt | Weight percentage of the silt particles (0.0002–0.05 mm), % |
| **bio17** | **Precipitation of Driest Quarter, mm** | **snd** | **Weight percentage of the sand particles (0.05–2 mm), %** |

**References**

1. Thiers, B. New York Botanical Garden’s Virtual Herbarium. *Index Herbariorum: A global directory of public herbaria and associated staff* (2022). http://sweetgum.nybg.org/science/ih/ (accessed 2 October 2022).

2. Vasileva, A. N. *et al.* *Campanulaceae* in *Flora of Kazakhstan. VIII* (ed. Pavlov, N. V.), 288–302 (Nauka, 1965).

3. Abdusalamova, L. N. *et al.* *Campanulaceae* in *Frora of Tajikistan. IX* (eds. Ovchinnikov, P. N. & Kinzikaeva, G. K.), 148–170 (Nauka, 1988).

4. Shaw, J., Lickey, E. B., Schilling, E. E. & Small, R. L. Comparison of Whole Chloroplast Genome Sequences to Choose Noncoding Regions for Phylogenetic Studies in Angiosperms: The Tortoise and the Hare III. *Am. J. Bot.* **94**, 275–288. https://doi.org/10.3732/ajb.94.3.275 (2007).

5. Löhne, C., Borsch, T. & Wiersema, J. H. Phylogenetic Analysis of Nymphaeales Using Fast-Evolving and Noncoding Chloroplast Markers. *Bot. J. Linn. Soc.* **154**, 141–163. https://doi.org/10.1111/j.1095-8339.2007.00659.x (2007).

6. Campagna, M. L. & Downie, S. R. The Intron in Chloroplast Gene rpl16 is Missing From the Flowering Plant Families Geraniaceae, Goodeniaceae, and Plumbaginaceae. *Trans. Illinois State Acad. Sci.* **91**, 1–11 (1998).

7. Demesure, B., Sodzi, N. & Petit, R. J. A set of universal primers for amplification of polymorphic non-coding regions of mitochondrial and chloroplast DNA in plants. *Mol. Ecol.* **4**(1), 129–130. https://doi.org/10.1111/j.1365-294x.1995.tb00201.x (1995).

8. Löhne, T. & Borsch, C. Molecular evolution and phylogenetic utility of the petD group II intron: a case study in basal angiosperms. *Mol. Biol. Evol.* **22**(2), 317–332. https://doi.org/10.1093/molbev/msi019 (2005).

9. Wicke, S. & Quandt, D. Universal primers for the amplification of the plastid ‘trnK/matK’ region in land plants. *An. del Jardín Botánico Madrid* **66**(2), 285–288. https://doi.org/10.3989/ajbm.2231 (2009).

10. Shaw, J. *et al.* The tortoise and the hare II: relative utility of 21 noncoding chloroplast DNA sequences for phylogenetic analysis. *Am. J. Bot.* **92**(1), 142–166. https://doi.org/10.3732/ajb.92.1.142 (2005).
